# Supplementary material for: Murine endothelial serine palmitoyltransferase 1 (SPTLC1) is required for vascular development and systemic sphingolipid homeostasis
Source: eLife. 2022 Oct 5;11:e78861. doi: 10.7554/eLife.78861 (PMC9578713; doi:10.7554/eLife.78861)
Supplement: Figure 1—source data 2. [file elife-78861-fig1-data2.zip › Figure 1C/Blots with lane information.pptx]

## Slide 1
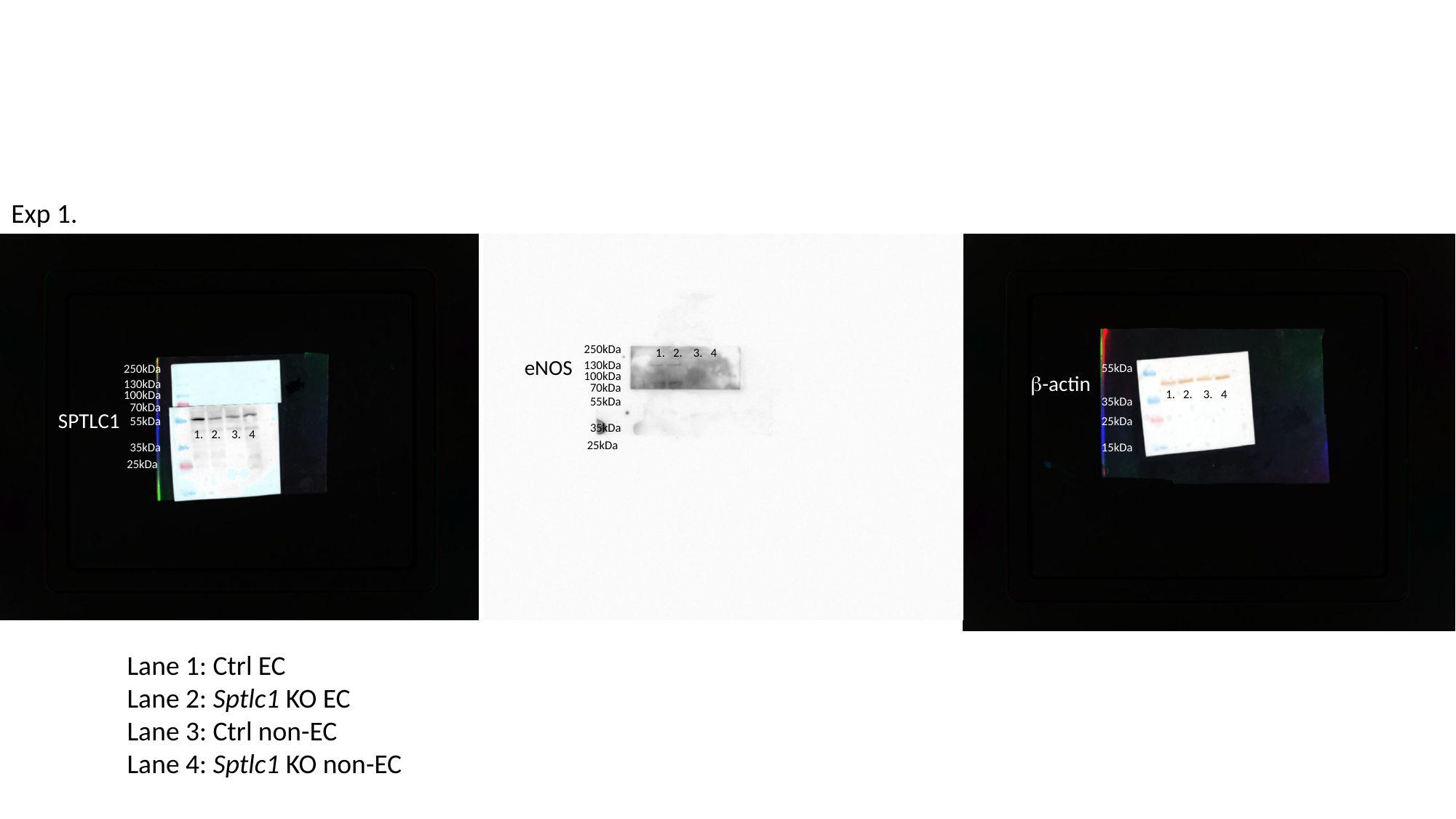

Exp 1.
250kDa
1. 2. 3. 4
eNOS
130kDa
55kDa
250kDa
100kDa
b-actin
130kDa
70kDa
1. 2. 3. 4
100kDa
55kDa
35kDa
70kDa
SPTLC1
55kDa
25kDa
35kDa
1. 2. 3. 4
25kDa
35kDa
15kDa
25kDa
Lane 1: Ctrl EC
Lane 2: Sptlc1 KO EC
Lane 3: Ctrl non-EC
Lane 4: Sptlc1 KO non-EC

## Slide 2
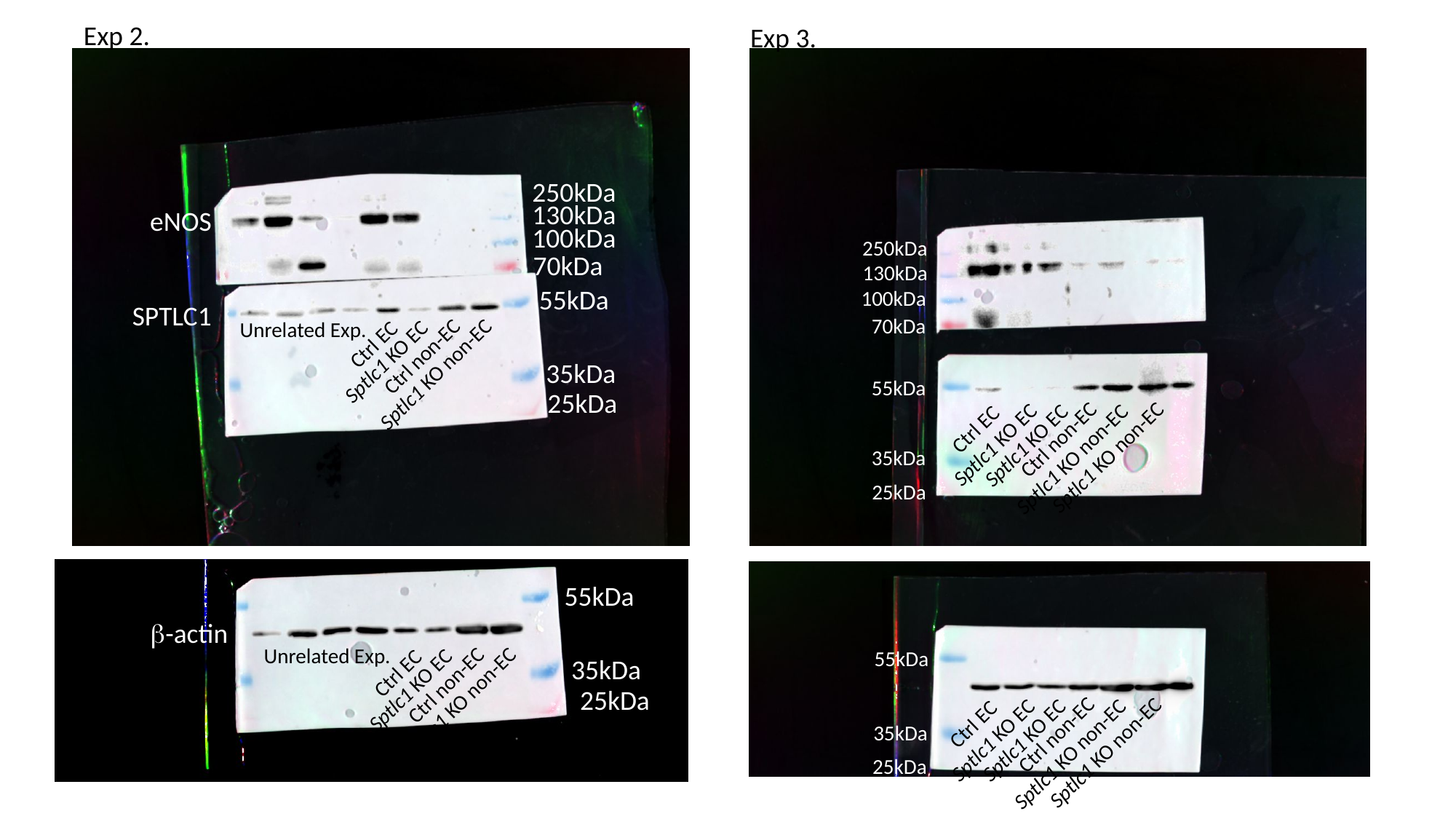

Exp 2.
Exp 3.
250kDa
130kDa
eNOS
100kDa
250kDa
70kDa
130kDa
55kDa
100kDa
SPTLC1
70kDa
Unrelated Exp.
Ctrl EC
Ctrl non-EC
Sptlc1 KO EC
35kDa
Sptlc1 KO non-EC
55kDa
25kDa
Ctrl EC
Ctrl non-EC
Sptlc1 KO EC
Sptlc1 KO EC
Sptlc1 KO non-EC
35kDa
Sptlc1 KO non-EC
25kDa
55kDa
b-actin
Unrelated Exp.
55kDa
35kDa
Ctrl EC
Ctrl non-EC
Sptlc1 KO EC
25kDa
Sptlc1 KO non-EC
Ctrl EC
35kDa
Ctrl non-EC
Sptlc1 KO EC
Sptlc1 KO EC
Sptlc1 KO non-EC
Sptlc1 KO non-EC
25kDa
