# Supplementary figures and images for: Murine endothelial serine palmitoyltransferase 1 (SPTLC1) is required for vascular development and systemic sphingolipid homeostasis
Source: eLife. 2022 Oct 5;11:e78861. doi: 10.7554/eLife.78861 (PMC9578713; doi:10.7554/eLife.78861)

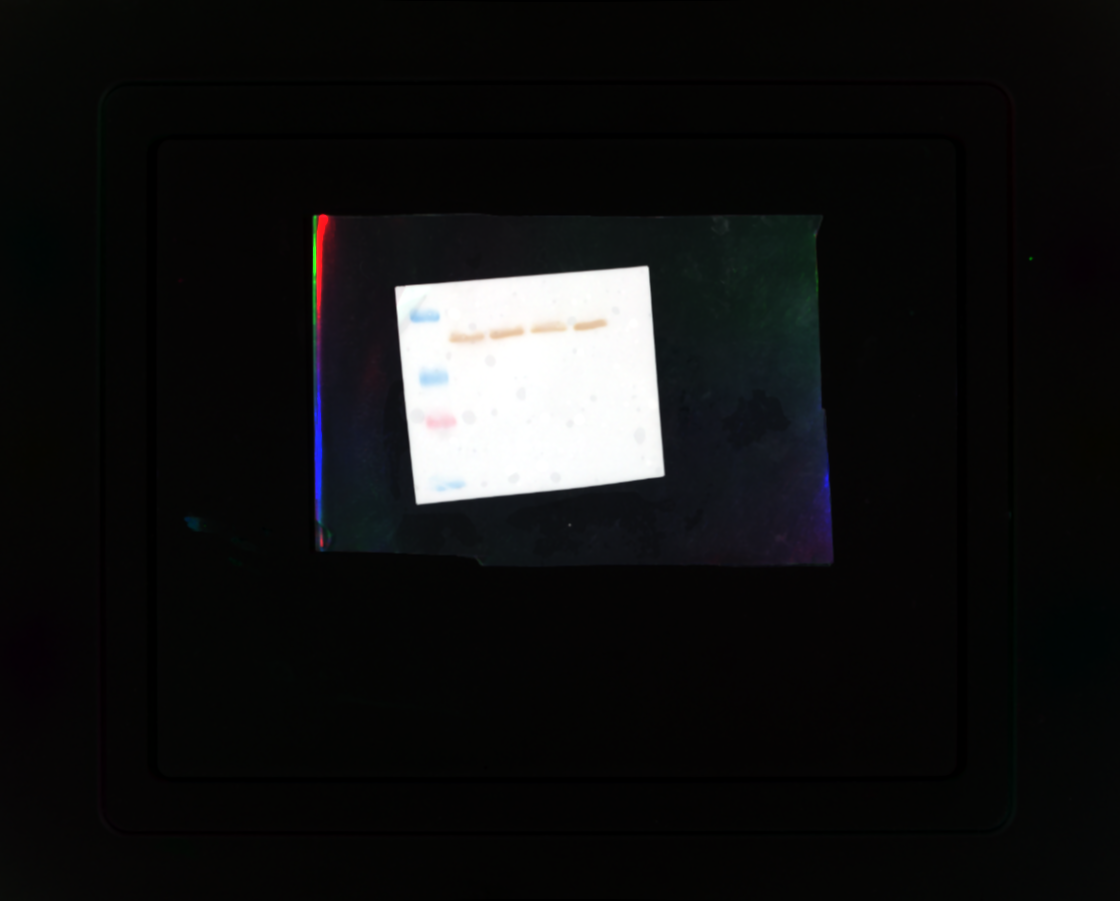

Supplement: Figure 1—source data 2. [file elife-78861-fig1-data2.zip › Figure 1C/Exp 1/Exp1 Actin.tif]

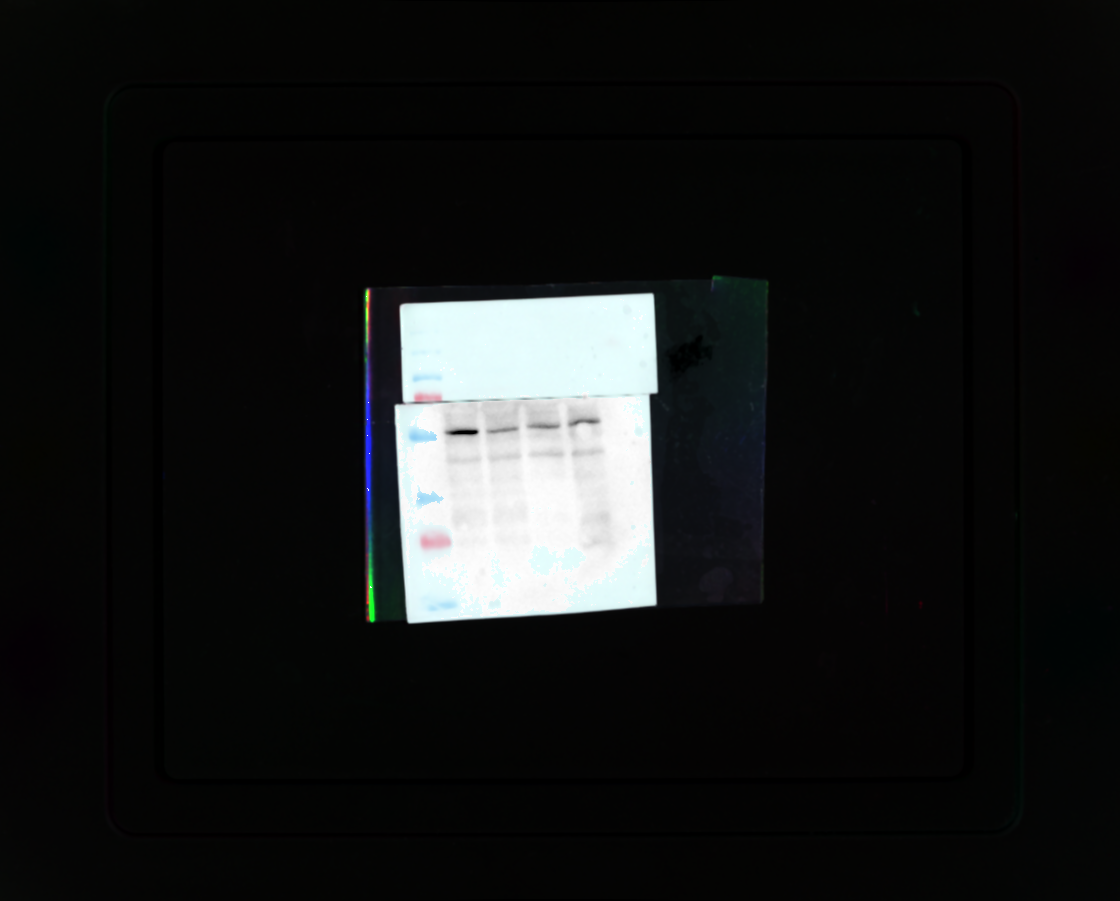

Supplement: Figure 1—source data 2. [file elife-78861-fig1-data2.zip › Figure 1C/Exp 1/Exp1 SPTLC1.tif]

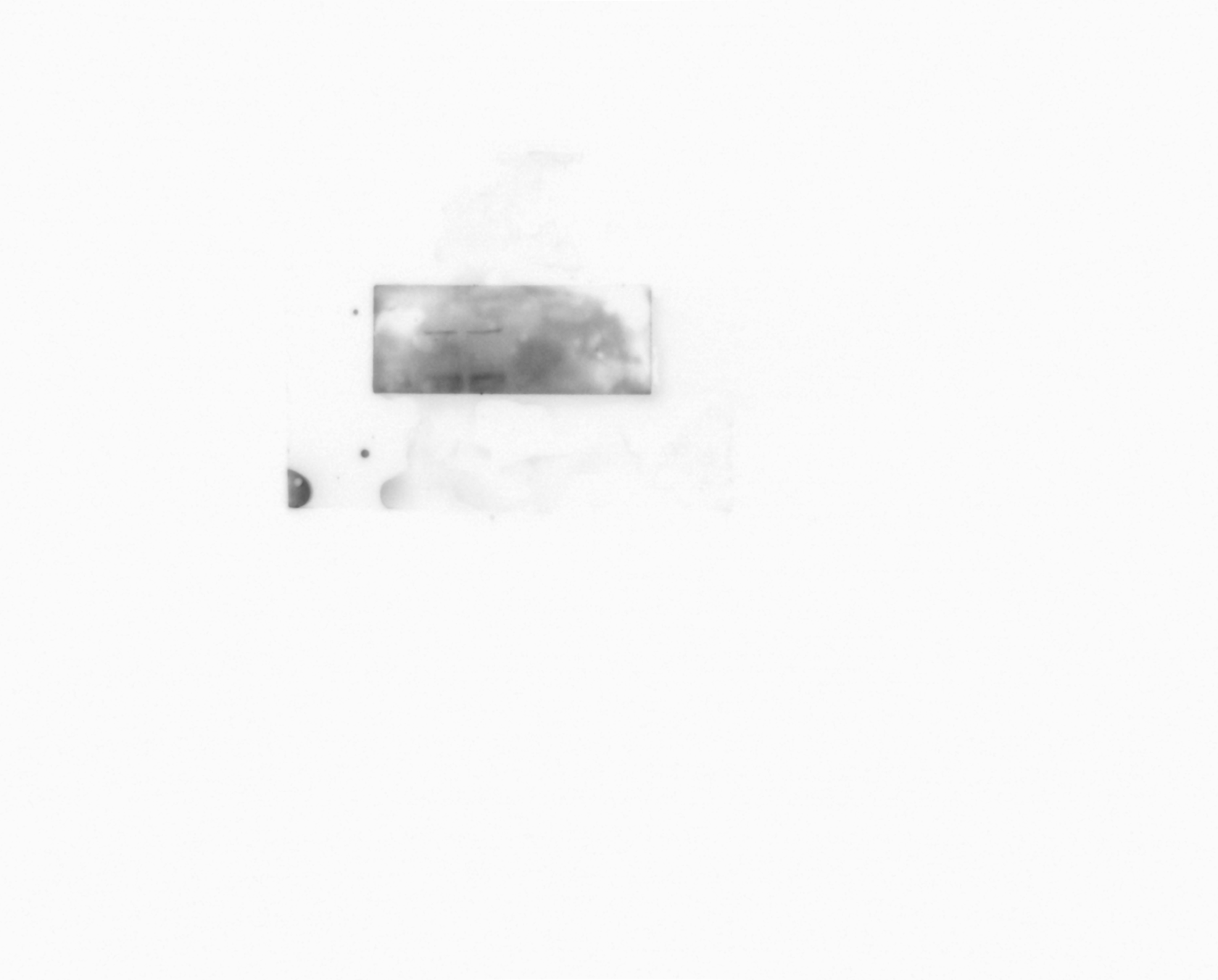

Supplement: Figure 1—source data 2. [file elife-78861-fig1-data2.zip › Figure 1C/Exp 1/Exp1 eNOS.tif]

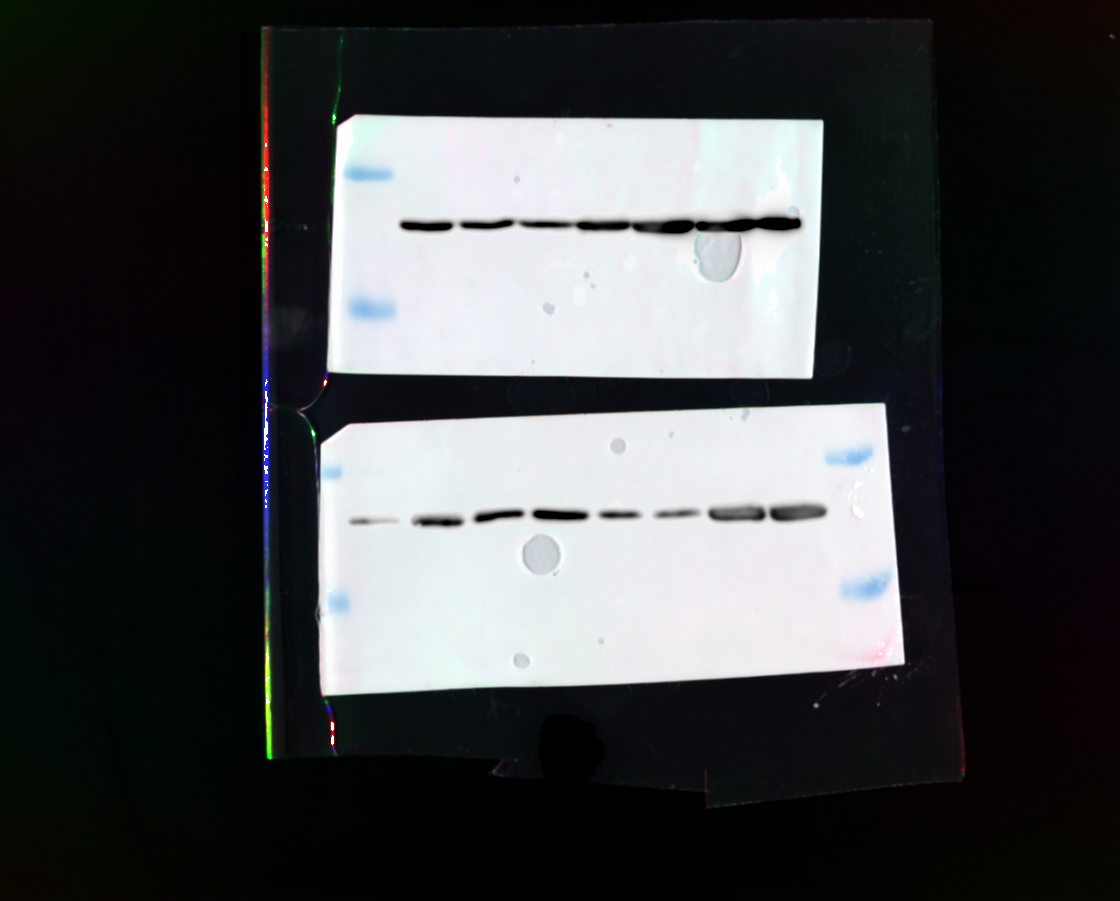

Supplement: Figure 1—source data 2. [file elife-78861-fig1-data2.zip › Figure 1C/Exp 2+3/Exp2 + Exp3 Actin.tif]

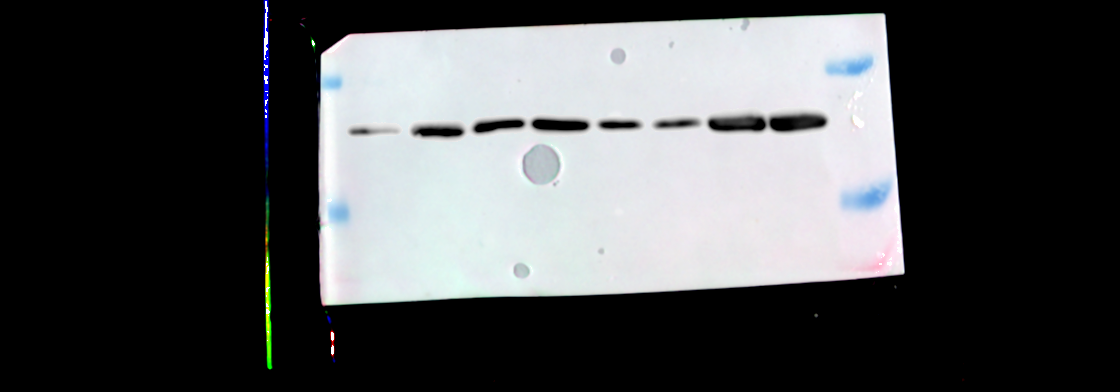

Supplement: Figure 1—source data 2. [file elife-78861-fig1-data2.zip › Figure 1C/Exp 2+3/Exp2 Actin.tif]

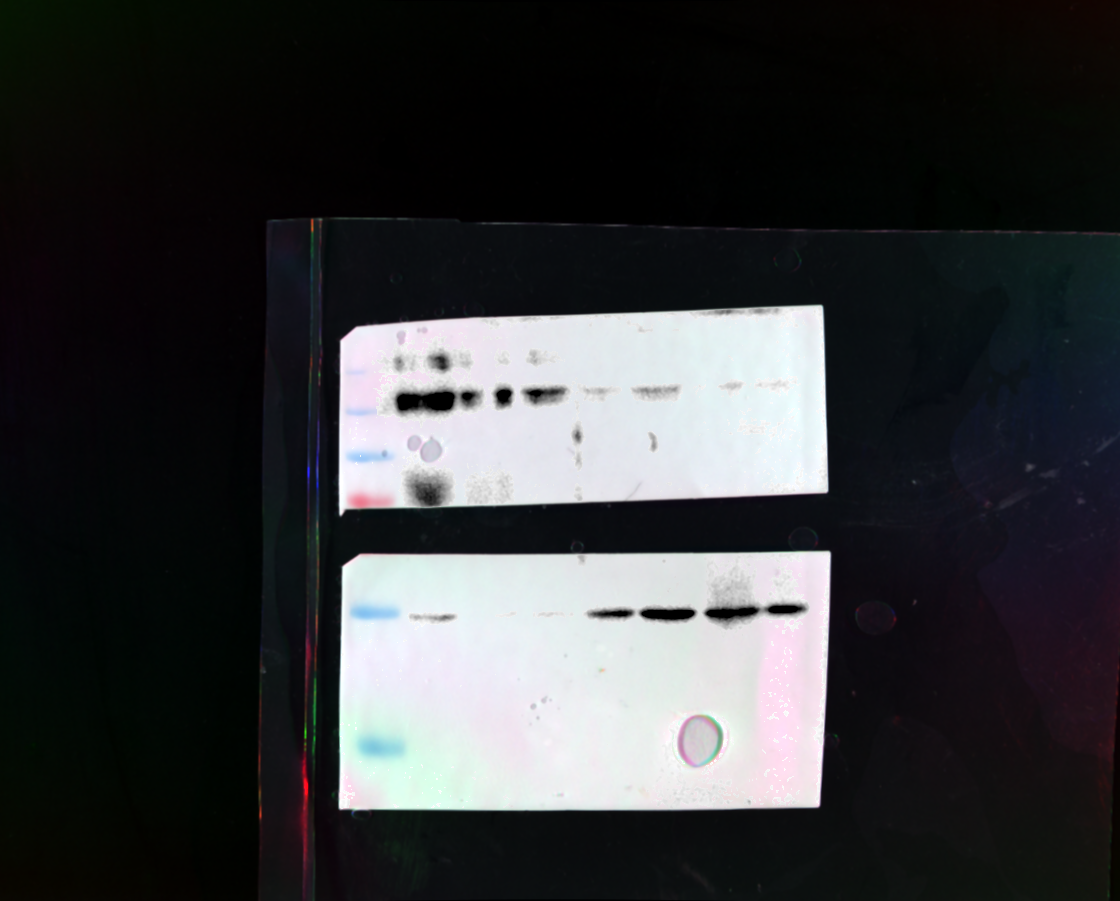

Supplement: Figure 1—source data 2. [file elife-78861-fig1-data2.zip › Figure 1C/Exp 2+3/Exp4 eNOS SPTLC1.tif]

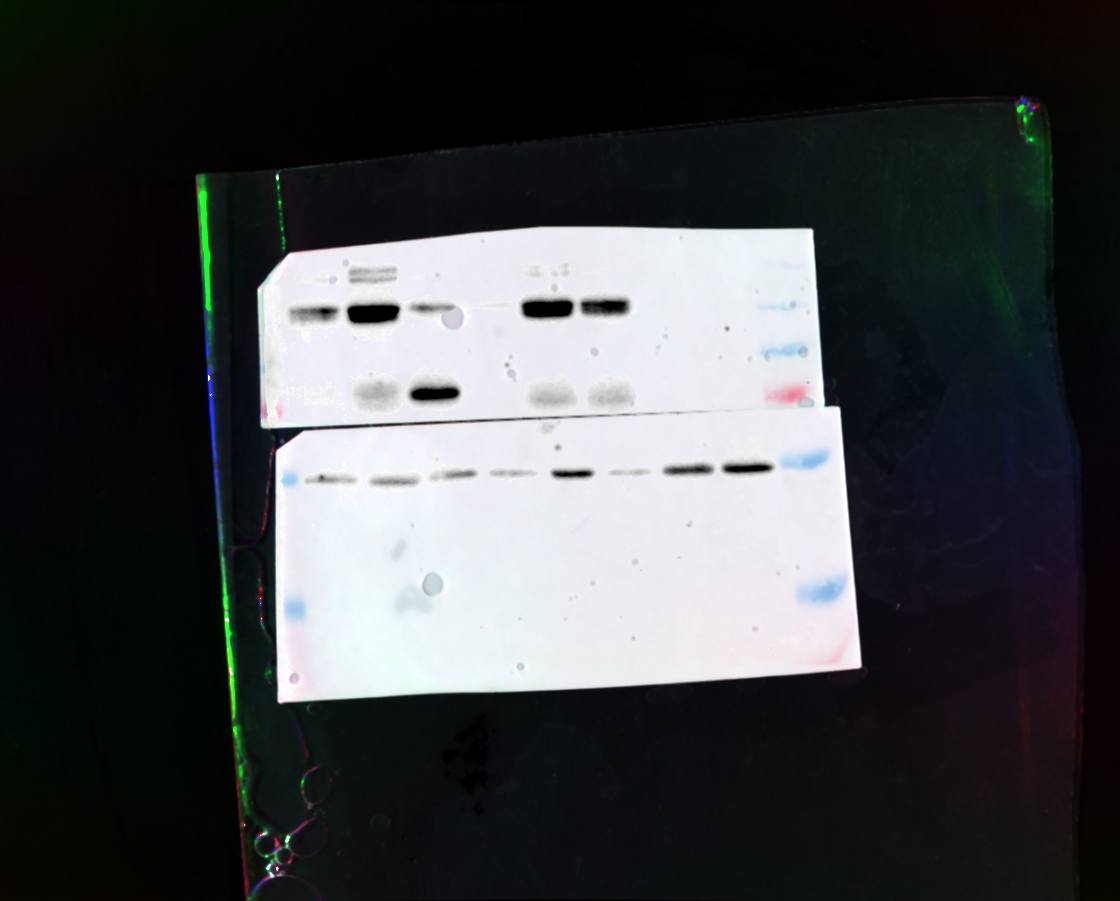

Supplement: Figure 1—source data 2. [file elife-78861-fig1-data2.zip › Figure 1C/Exp 2+3/Exp 2 eNOS SPTLC1.tif]

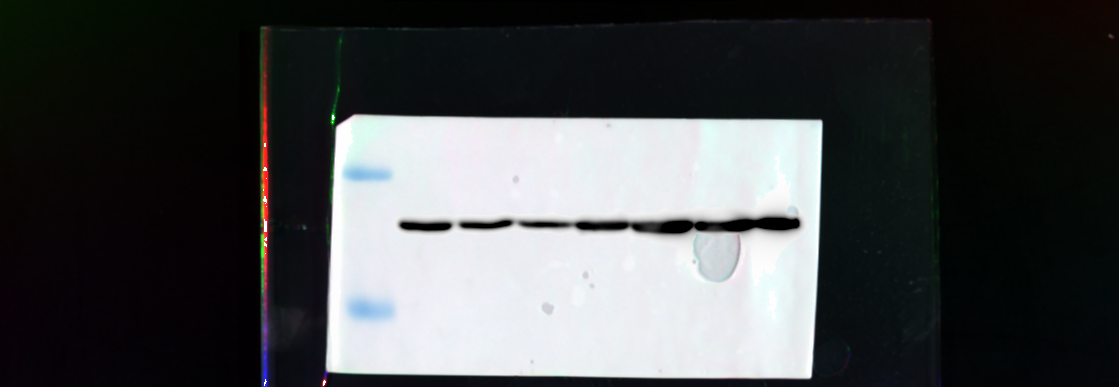

Supplement: Figure 1—source data 2. [file elife-78861-fig1-data2.zip › Figure 1C/Exp 2+3/Exp3 Actin.tif]

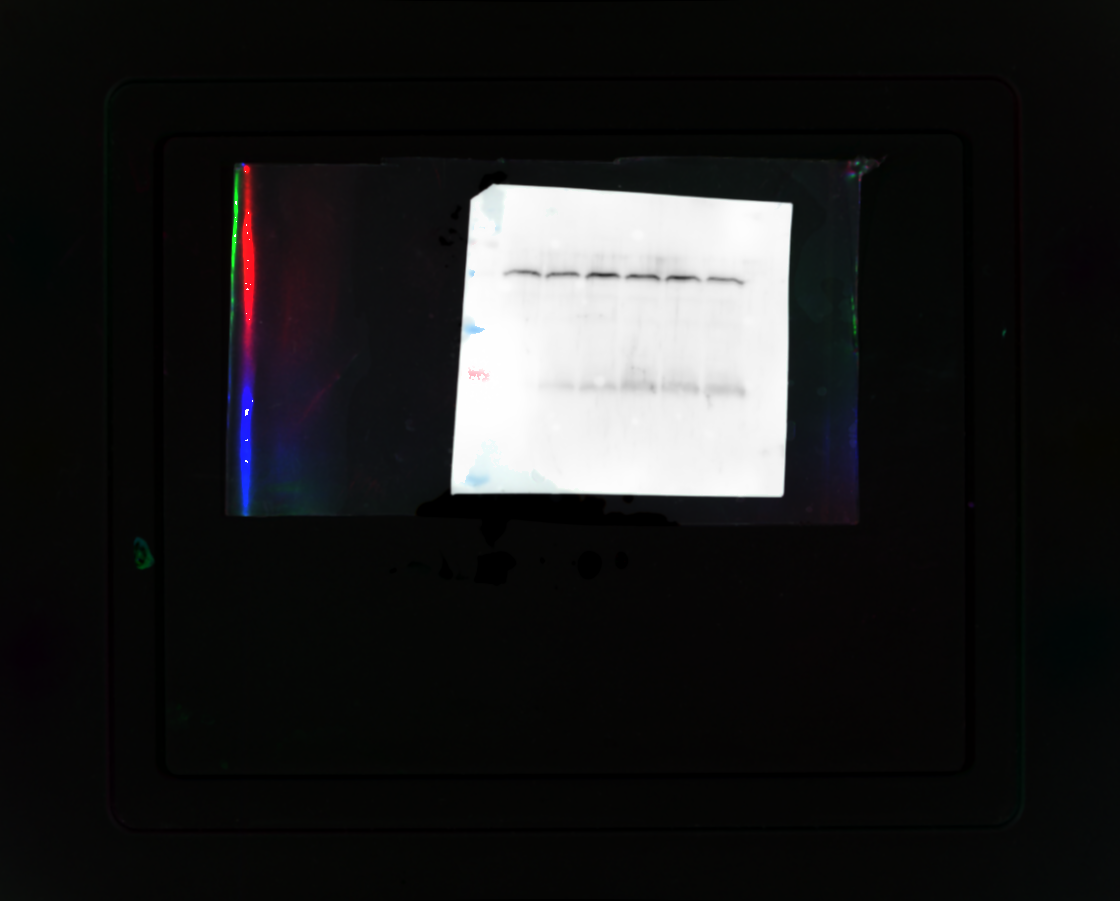

Supplement: Figure 1—figure supplement 2—source data 1. [file elife-78861-fig1-figsupp2-data1.zip › Figure 1 - Supplement 2 - Source Data/SPTLC1.tif]

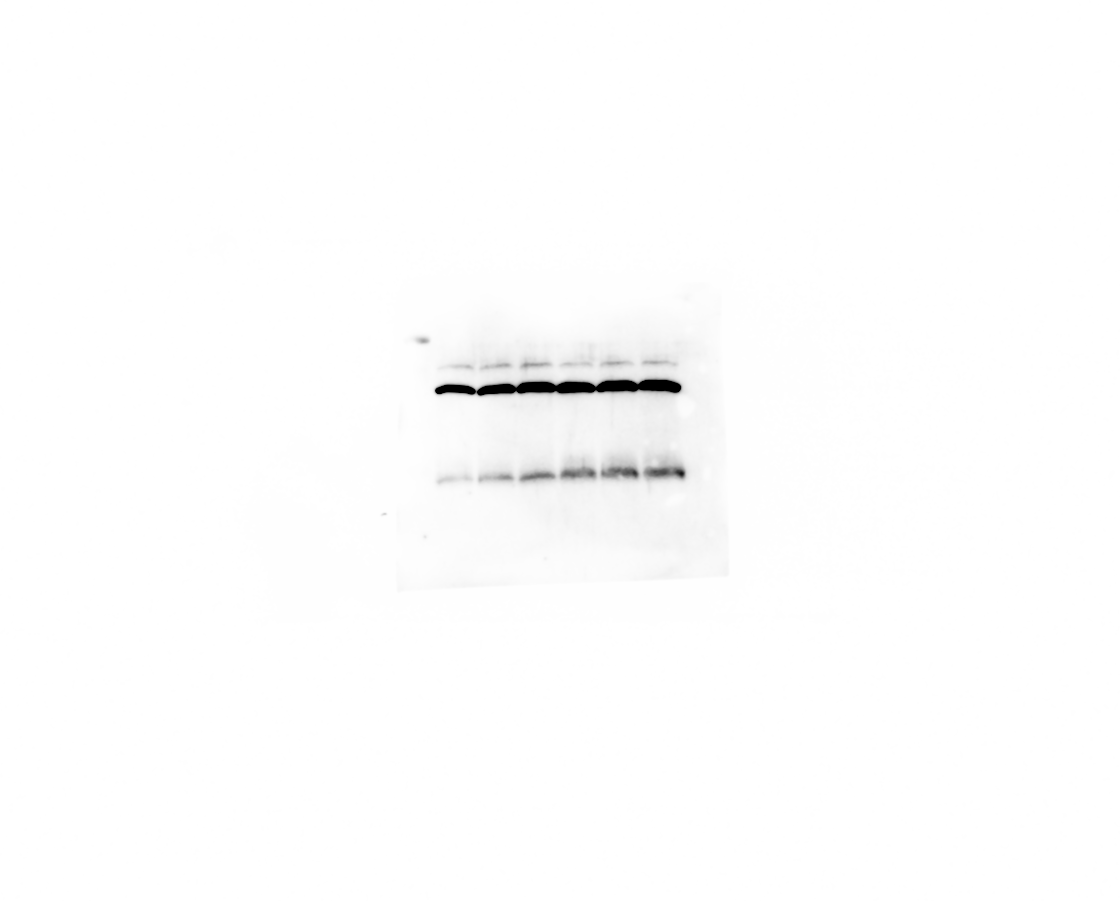

Supplement: Figure 1—figure supplement 2—source data 1. [file elife-78861-fig1-figsupp2-data1.zip › Figure 1 - Supplement 2 - Source Data/Actin.tif]

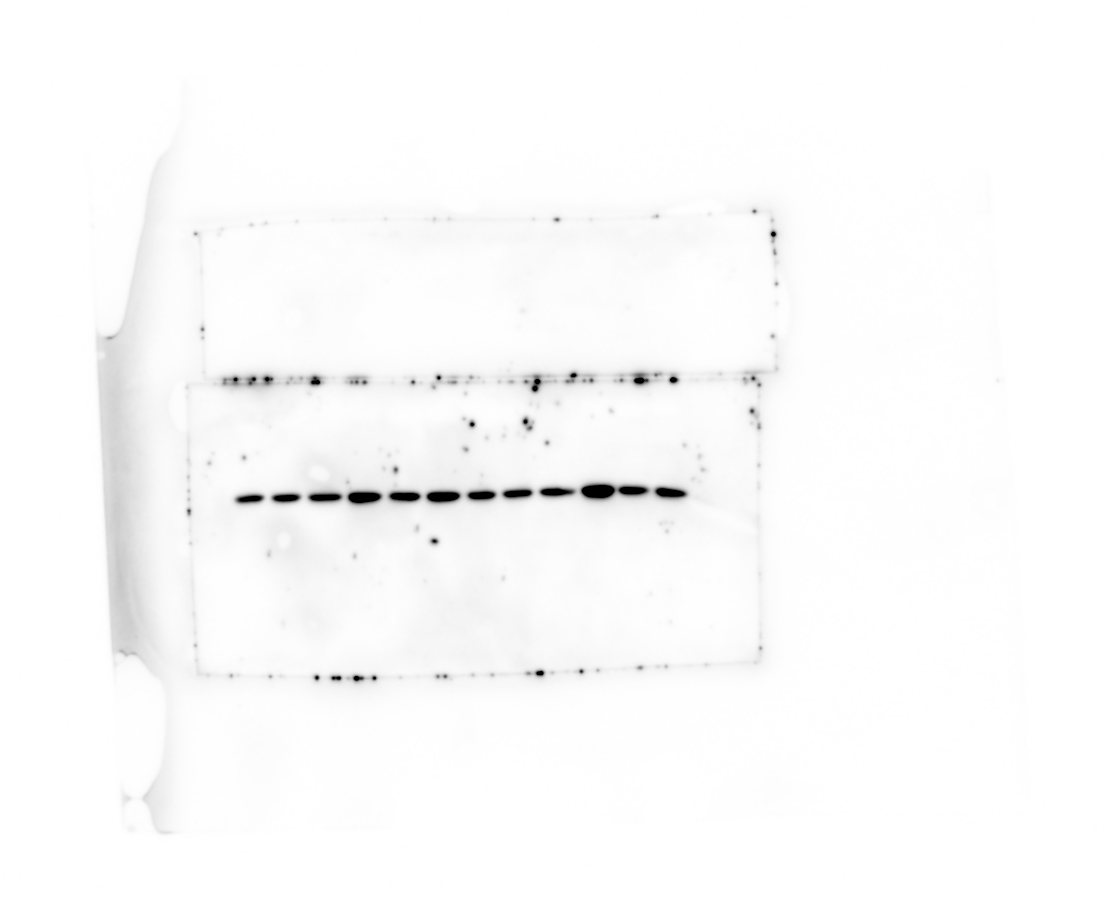

Supplement: Figure 1—figure supplement 4—source data 1. [file elife-78861-fig1-figsupp4-data1.zip › Figure 1 - Supplement 4/p-eNOS and b-actin.tif]

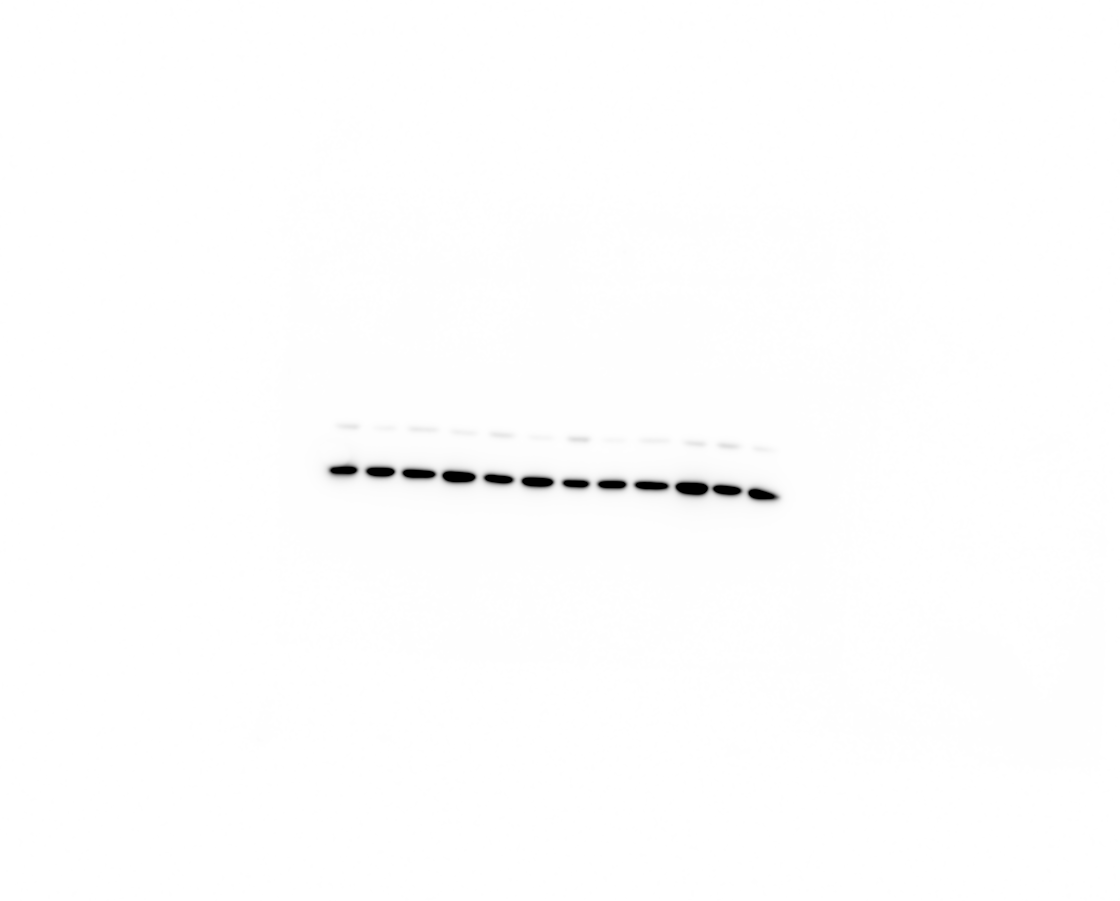

Supplement: Figure 1—figure supplement 4—source data 1. [file elife-78861-fig1-figsupp4-data1.zip › Figure 1 - Supplement 4/Total eNOS and SPTLC1.tif]

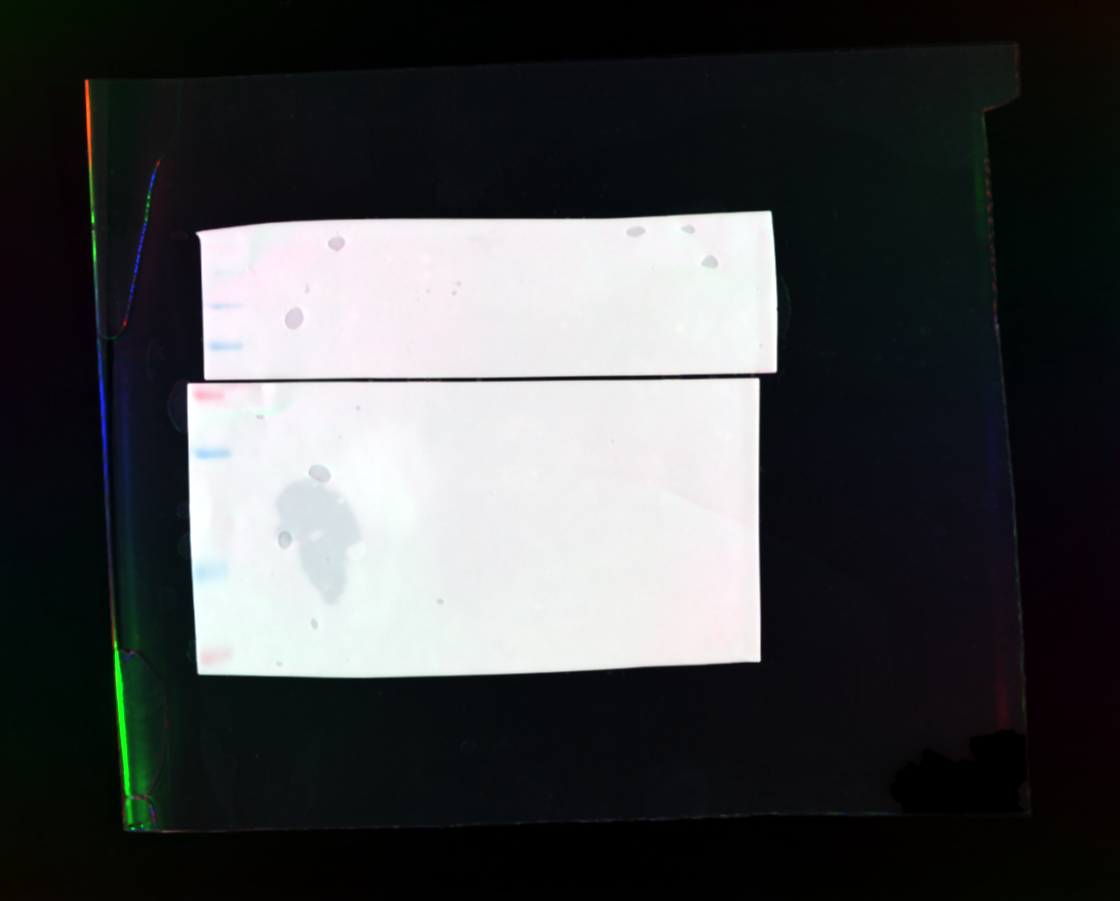

Supplement: Figure 1—figure supplement 4—source data 1. [file elife-78861-fig1-figsupp4-data1.zip › Figure 1 - Supplement 4/p-eNOS and b-actin Marker.tif]

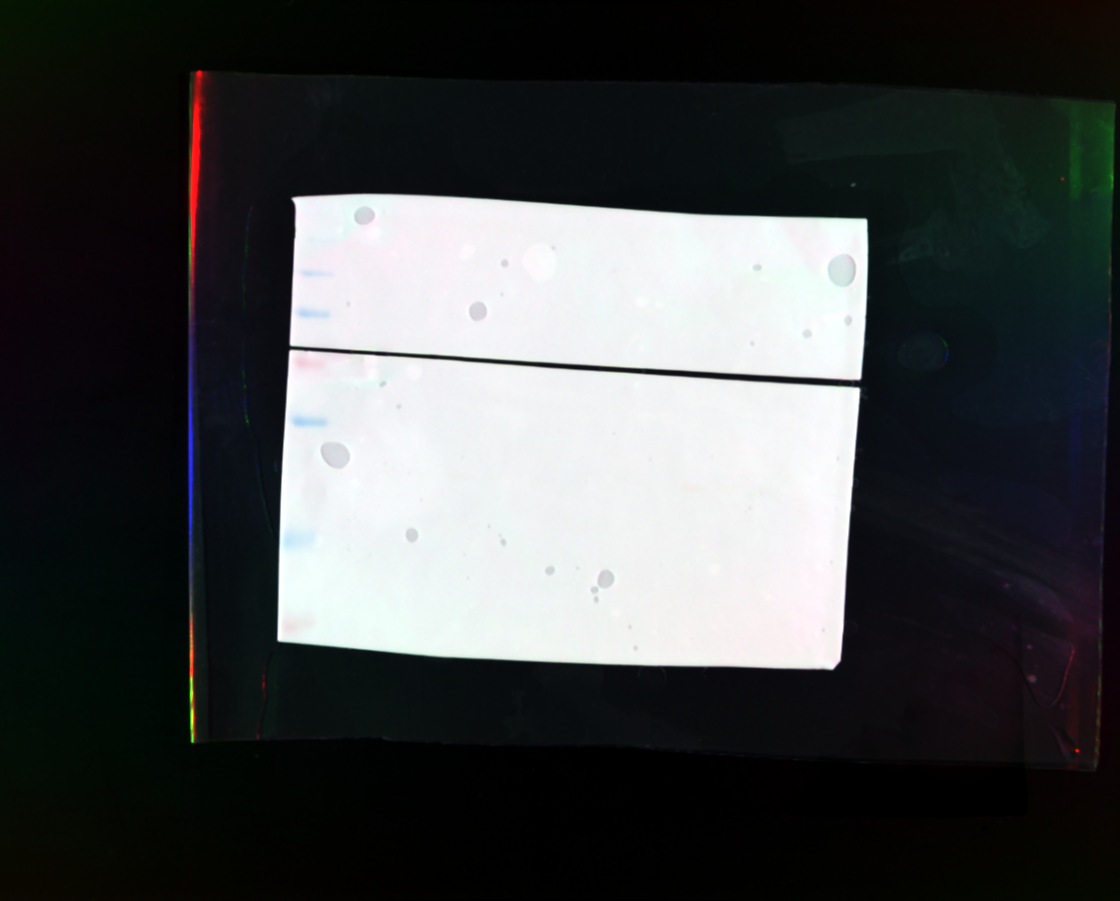

Supplement: Figure 1—figure supplement 4—source data 1. [file elife-78861-fig1-figsupp4-data1.zip › Figure 1 - Supplement 4/Total eNOS and SPTLC1 Marker.tif]

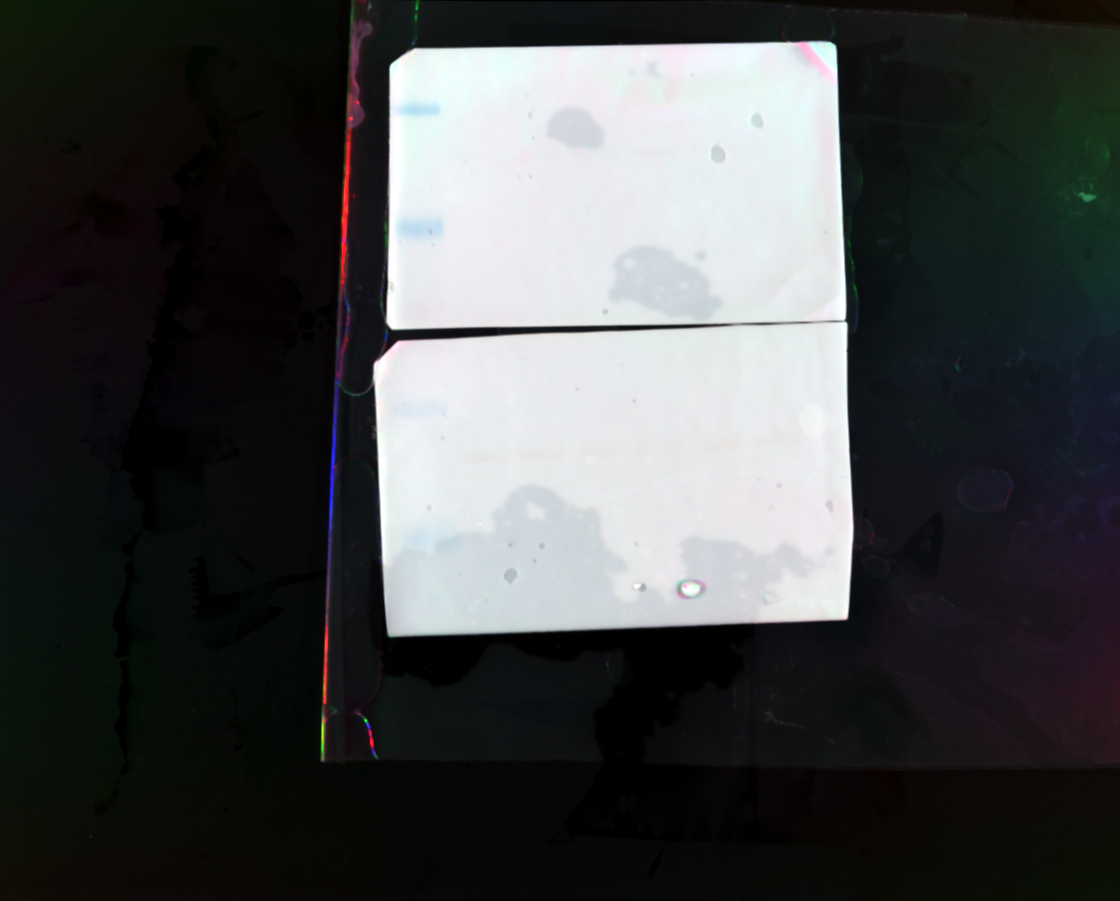

Supplement: Figure 5—source data 1. [file elife-78861-fig5-data1.zip › Figure 5D/Exp 3/Actin Marker.tif]

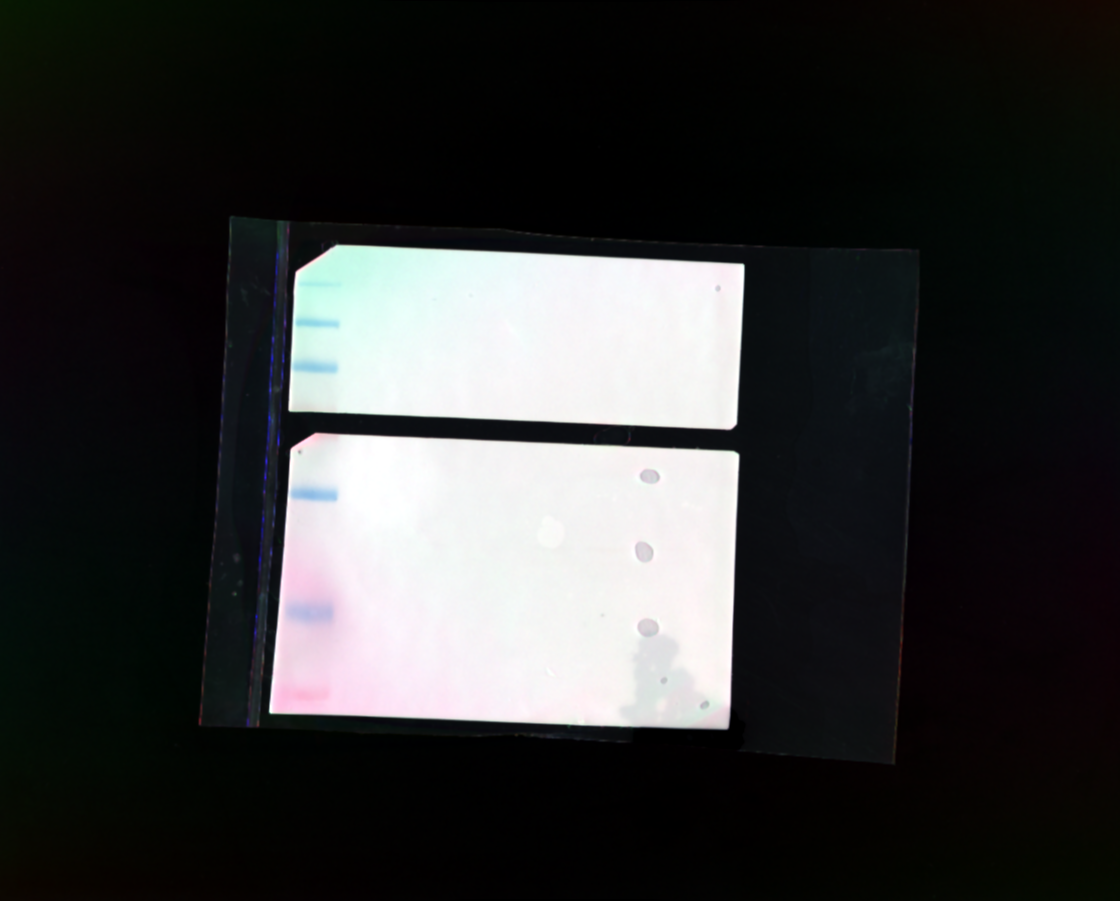

Supplement: Figure 5—source data 1. [file elife-78861-fig5-data1.zip › Figure 5D/Exp 3/Total ERK Marker.tif]

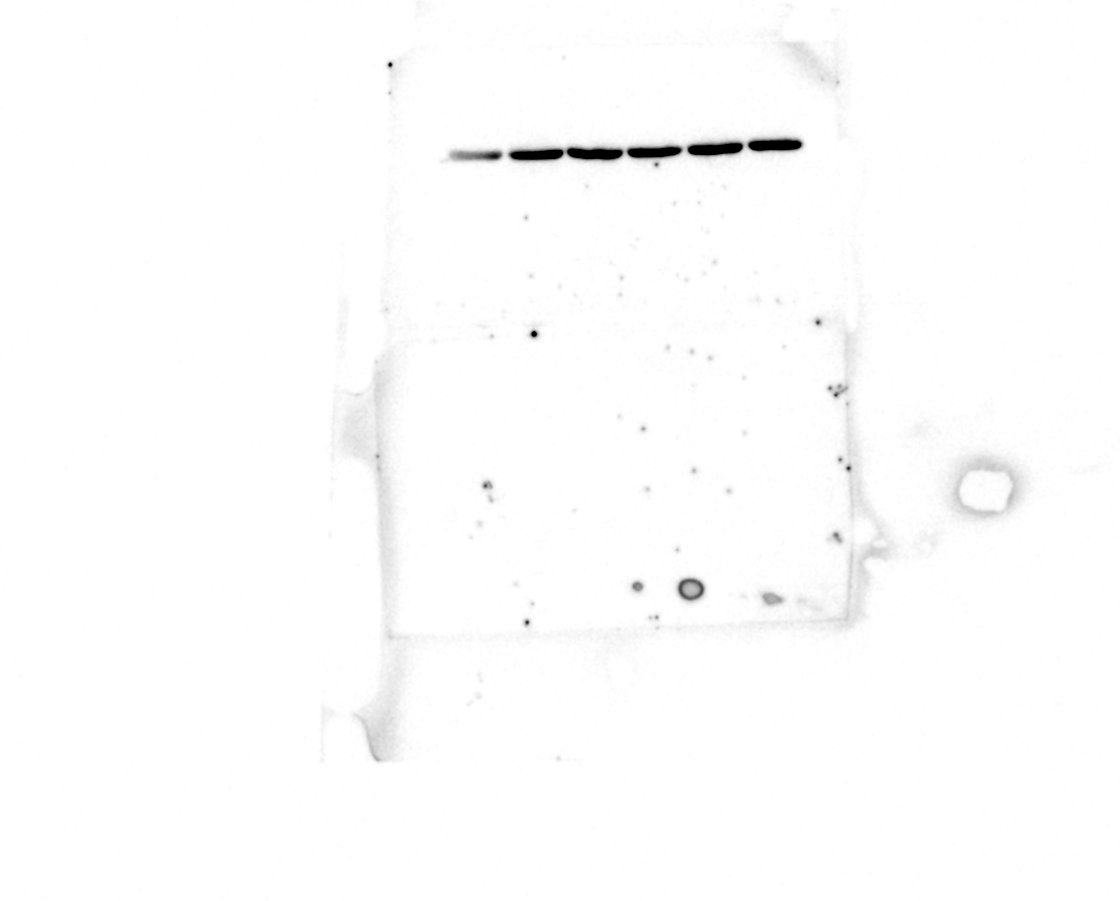

Supplement: Figure 5—source data 1. [file elife-78861-fig5-data1.zip › Figure 5D/Exp 3/Actin.tif]

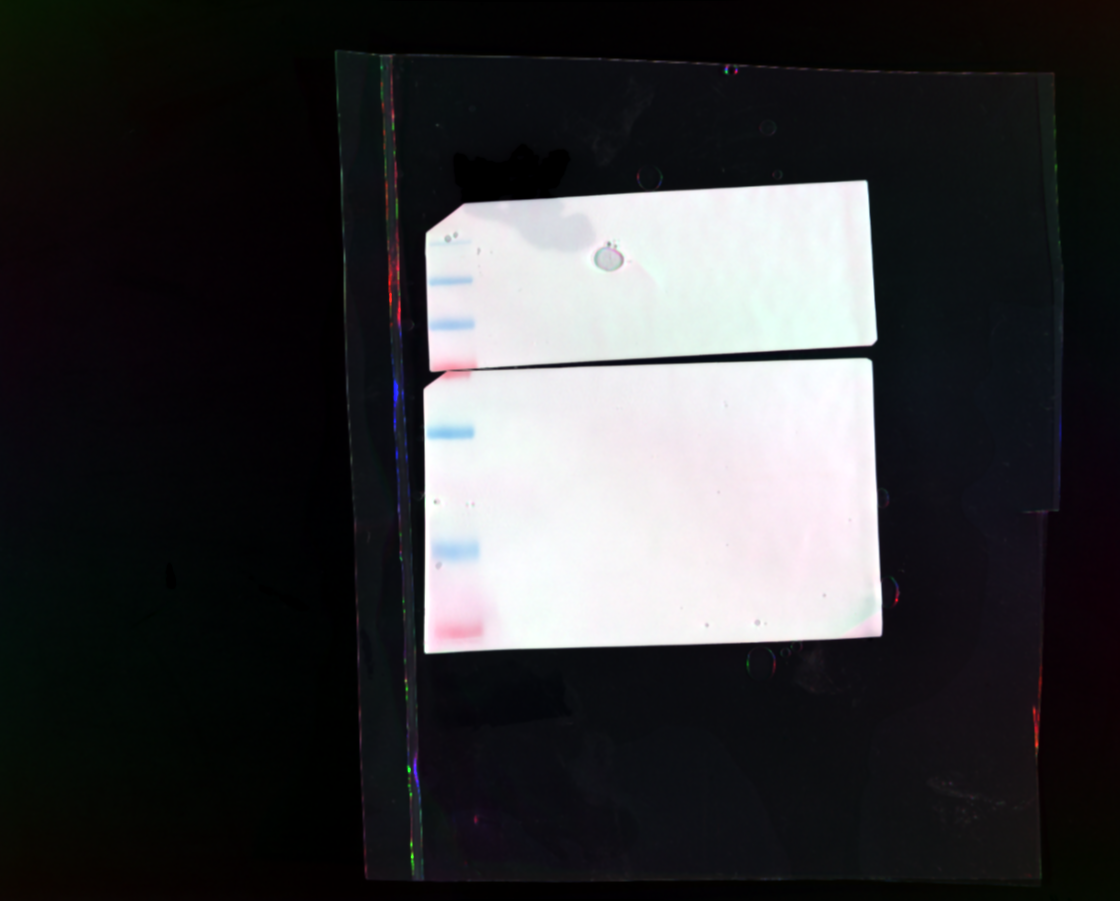

Supplement: Figure 5—source data 1. [file elife-78861-fig5-data1.zip › Figure 5D/Exp 3/Phospho-ERK Marker.tif]

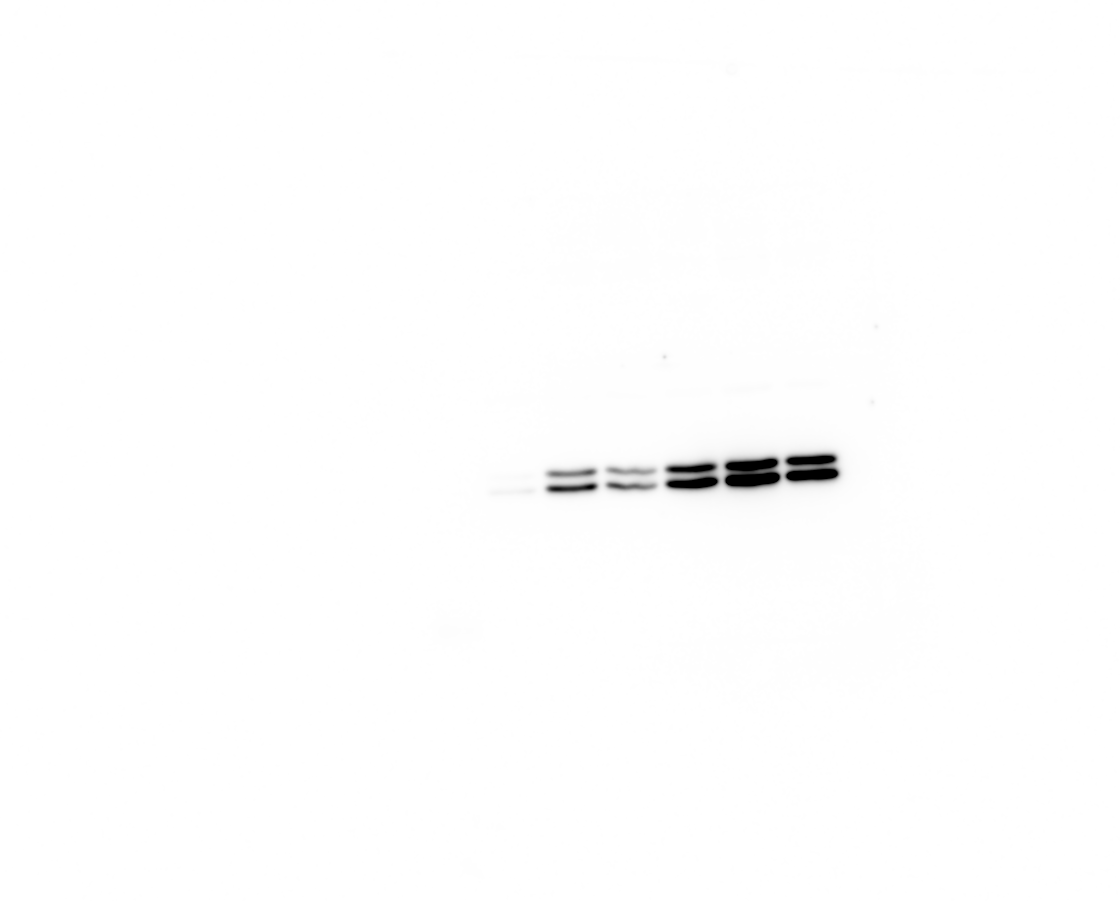

Supplement: Figure 5—source data 1. [file elife-78861-fig5-data1.zip › Figure 5D/Exp 3/Phospho-ERK.tif]

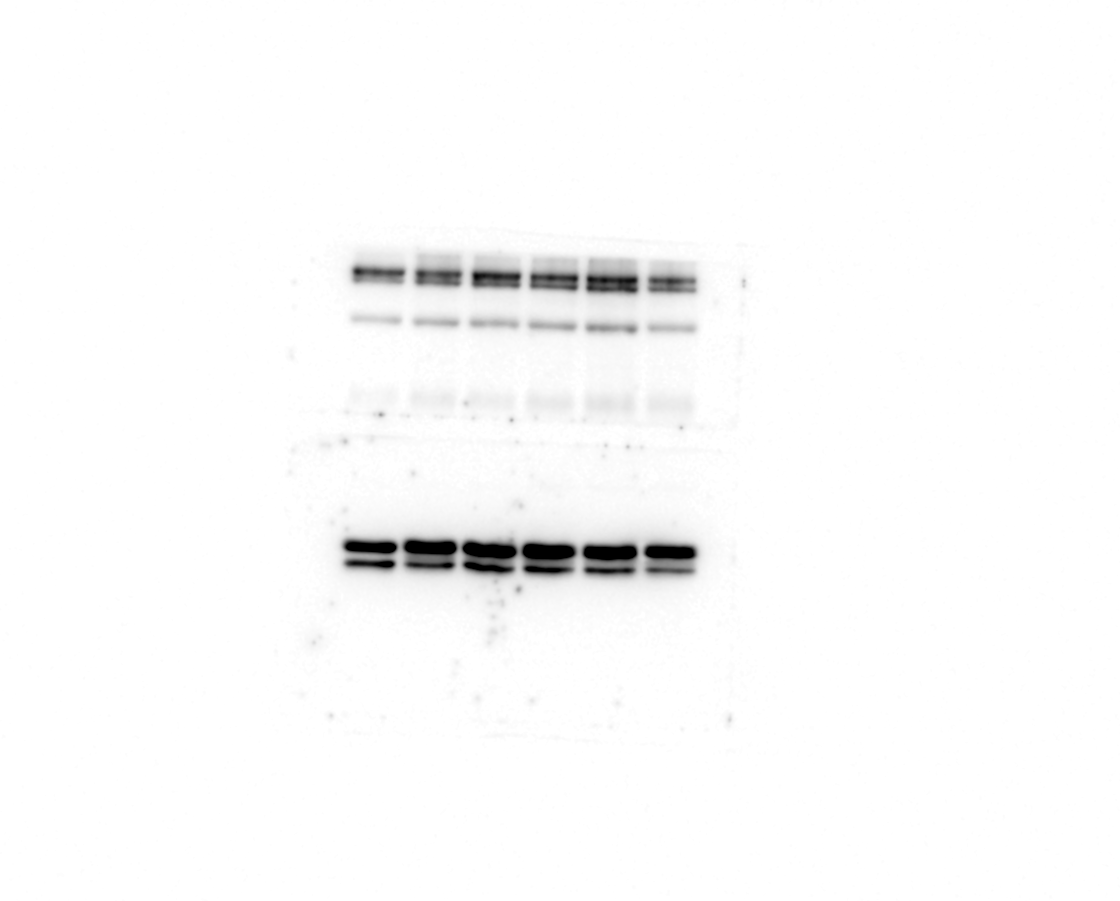

Supplement: Figure 5—source data 1. [file elife-78861-fig5-data1.zip › Figure 5D/Exp 3/Total ERK.tif]

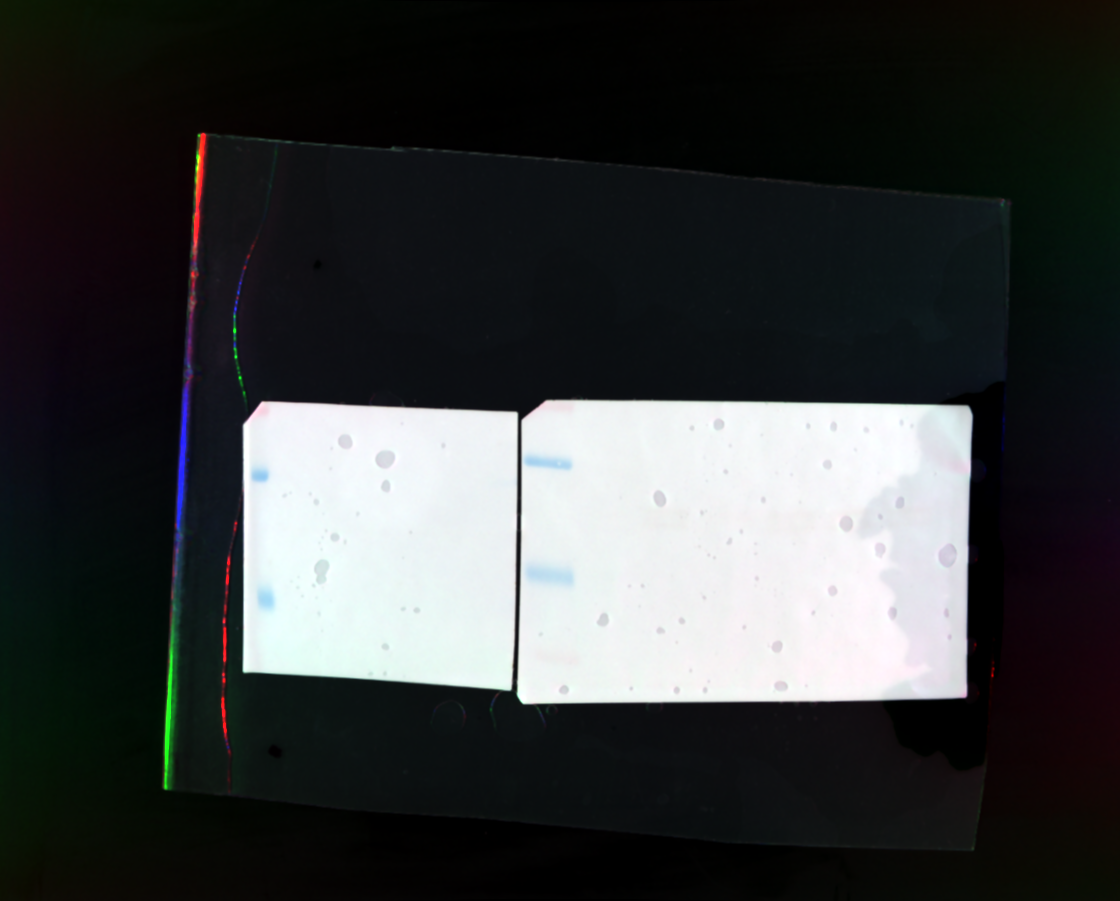

Supplement: Figure 5—source data 1. [file elife-78861-fig5-data1.zip › Figure 5D/Exp 4/Actin Marker.tif]

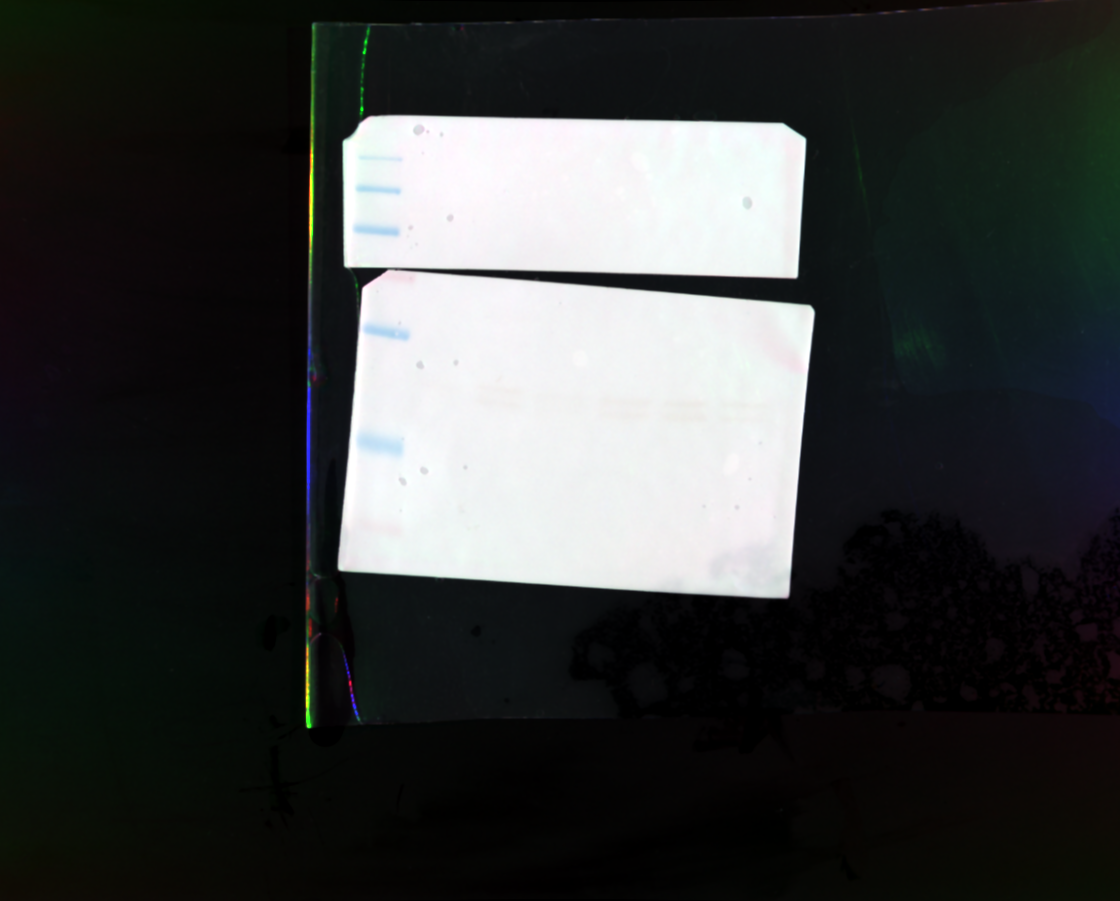

Supplement: Figure 5—source data 1. [file elife-78861-fig5-data1.zip › Figure 5D/Exp 4/Total ERK Marker.tif]

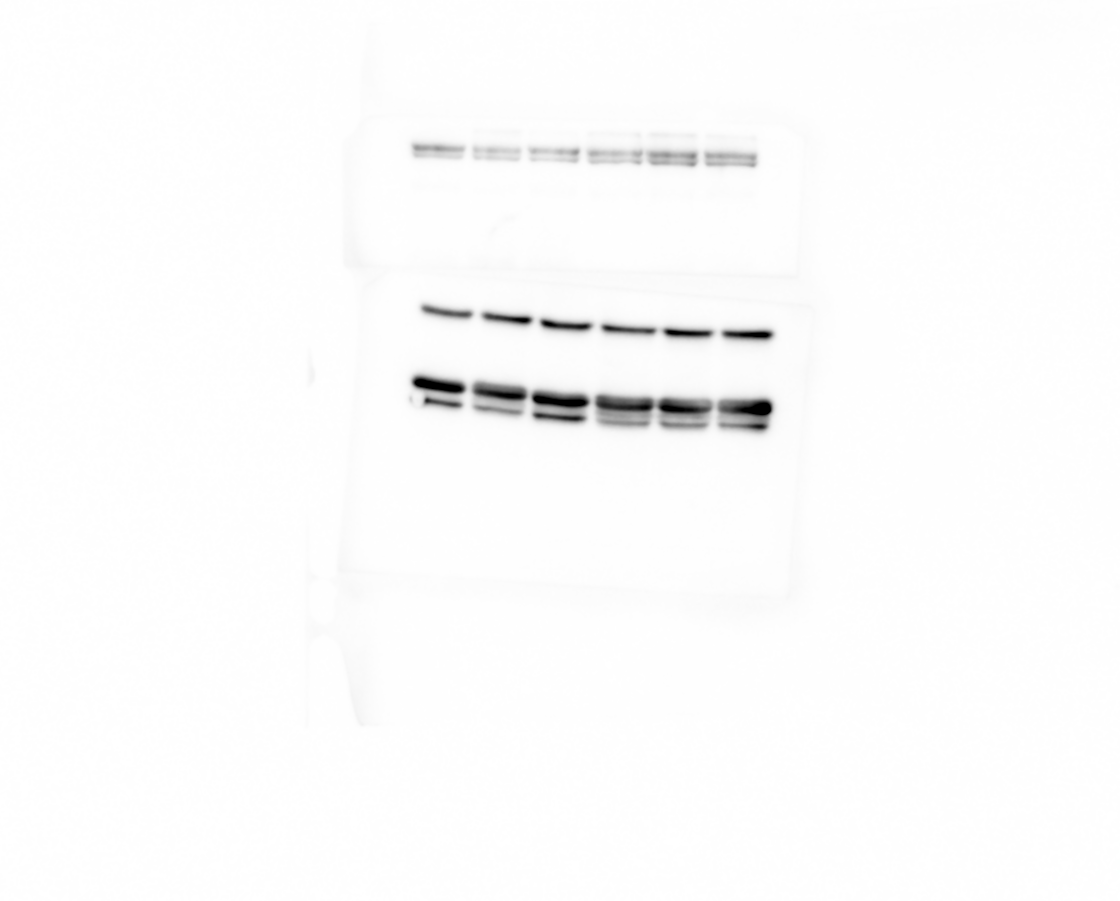

Supplement: Figure 5—source data 1. [file elife-78861-fig5-data1.zip › Figure 5D/Exp 4/Actin.tif]

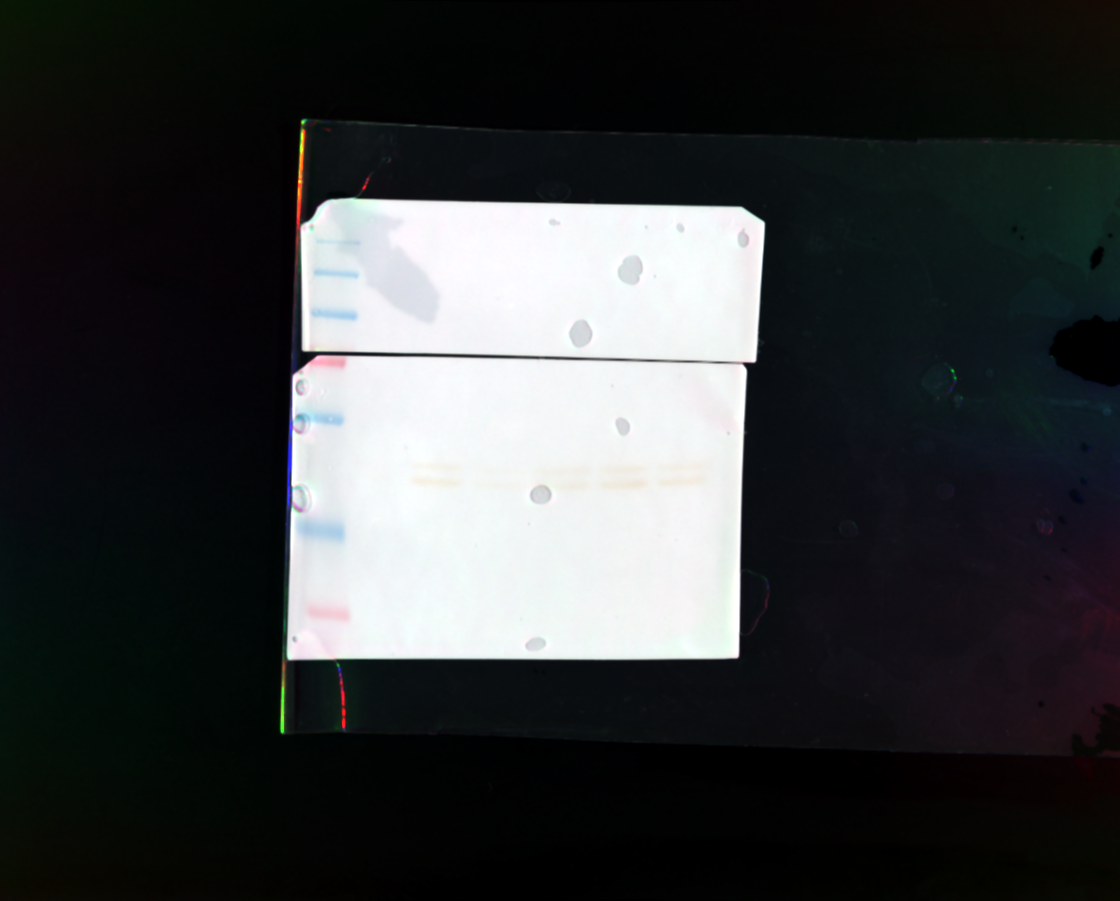

Supplement: Figure 5—source data 1. [file elife-78861-fig5-data1.zip › Figure 5D/Exp 4/Phospho-ERK Marker.tif]

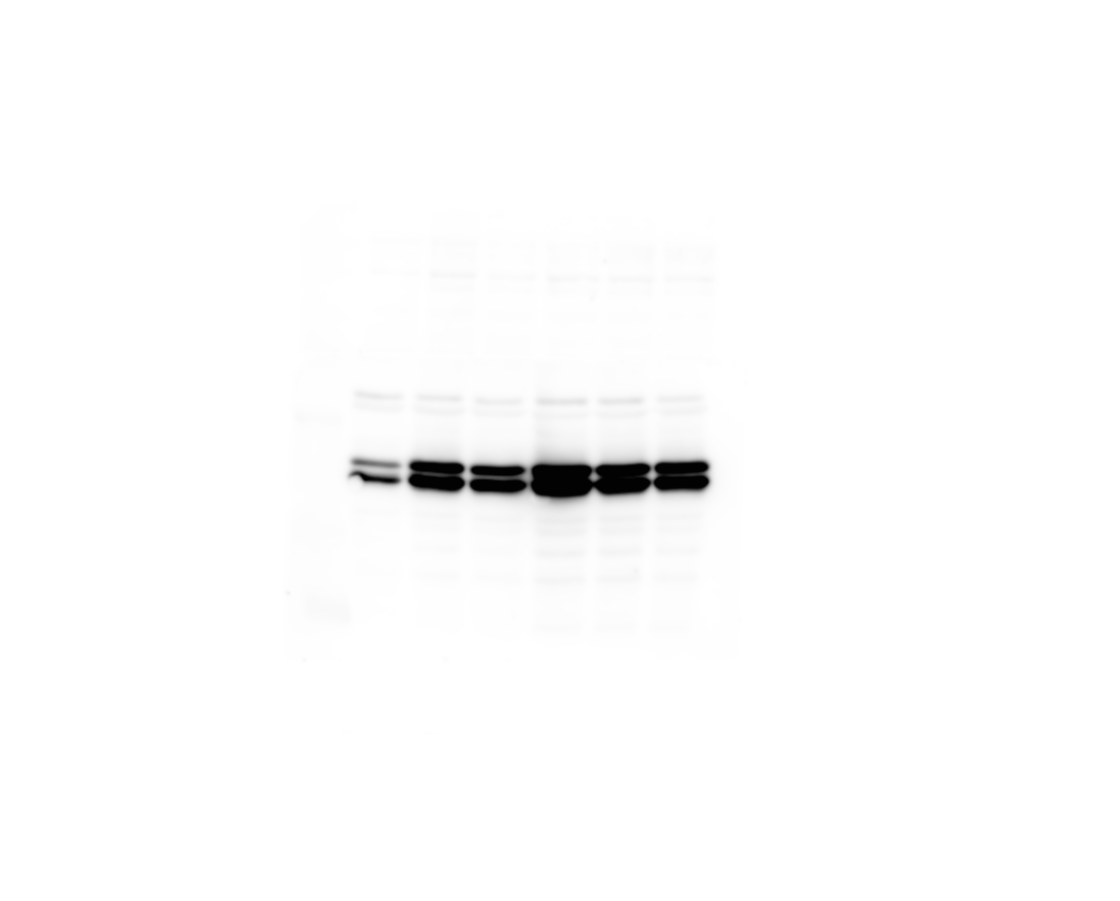

Supplement: Figure 5—source data 1. [file elife-78861-fig5-data1.zip › Figure 5D/Exp 4/Phospho-ERK.tif]

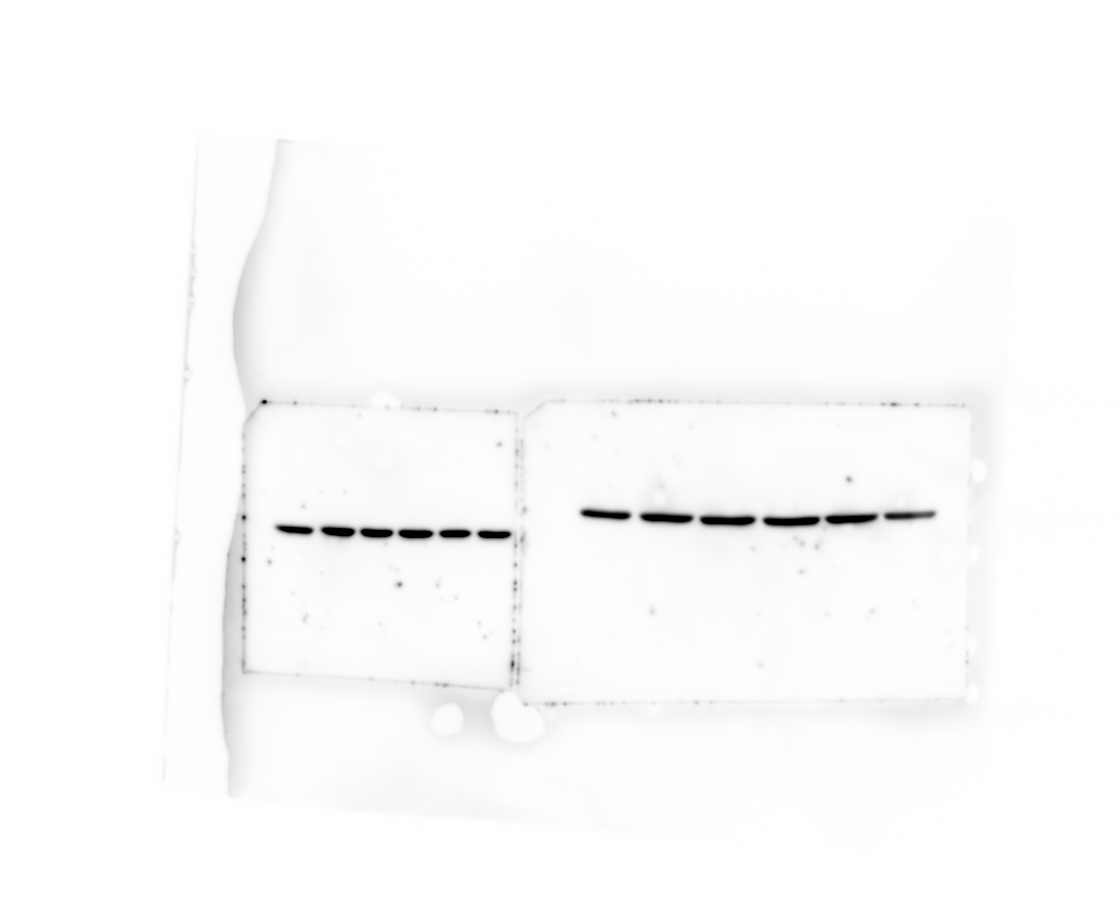

Supplement: Figure 5—source data 1. [file elife-78861-fig5-data1.zip › Figure 5D/Exp 4/Total ERK.tif]

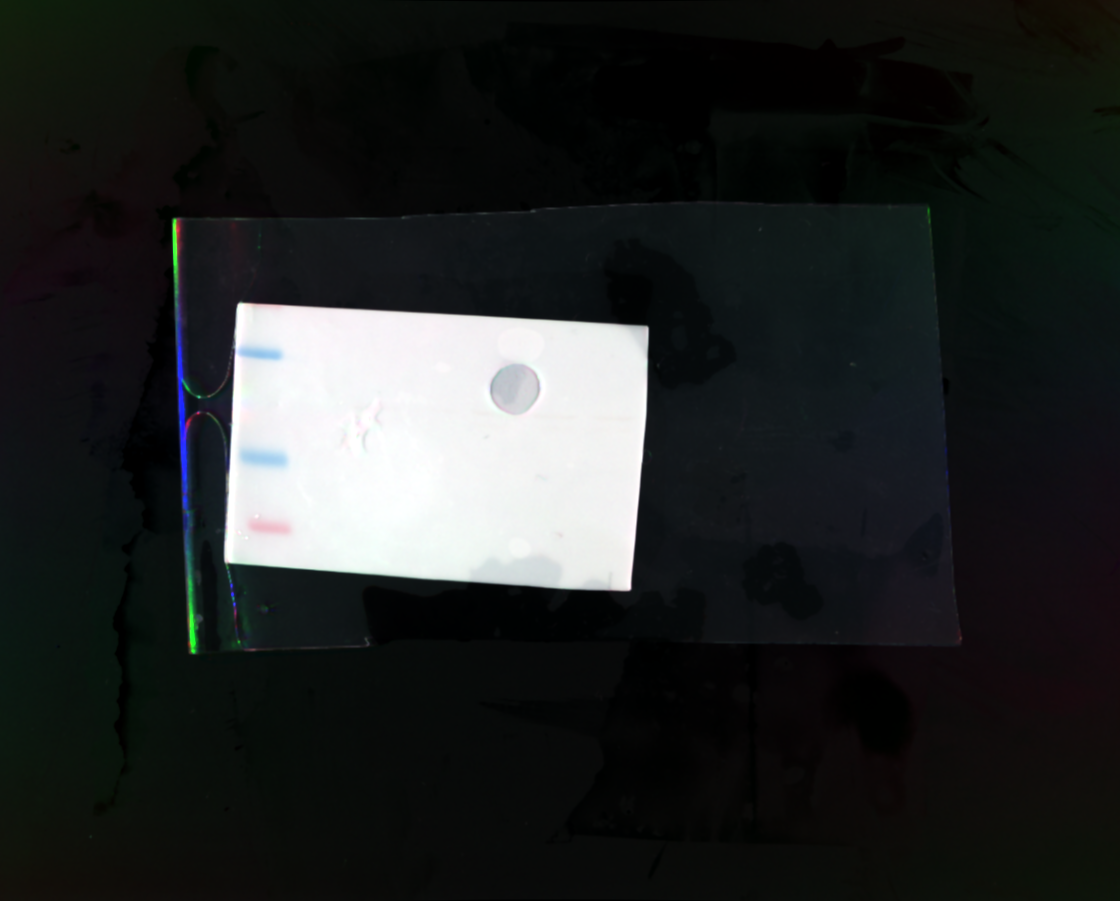

Supplement: Figure 5—source data 1. [file elife-78861-fig5-data1.zip › Figure 5D/Exp 2/Actin Marker.tif]

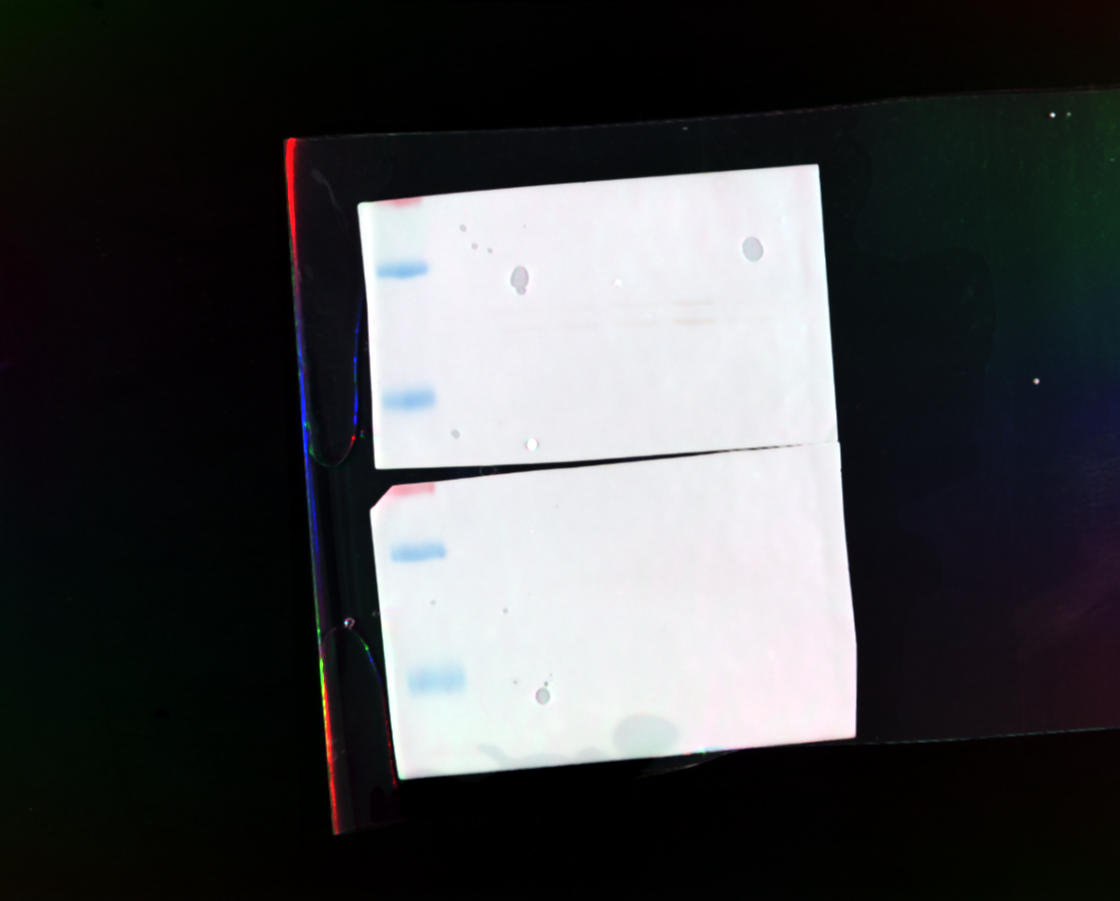

Supplement: Figure 5—source data 1. [file elife-78861-fig5-data1.zip › Figure 5D/Exp 2/Total ERK Marker.tif]

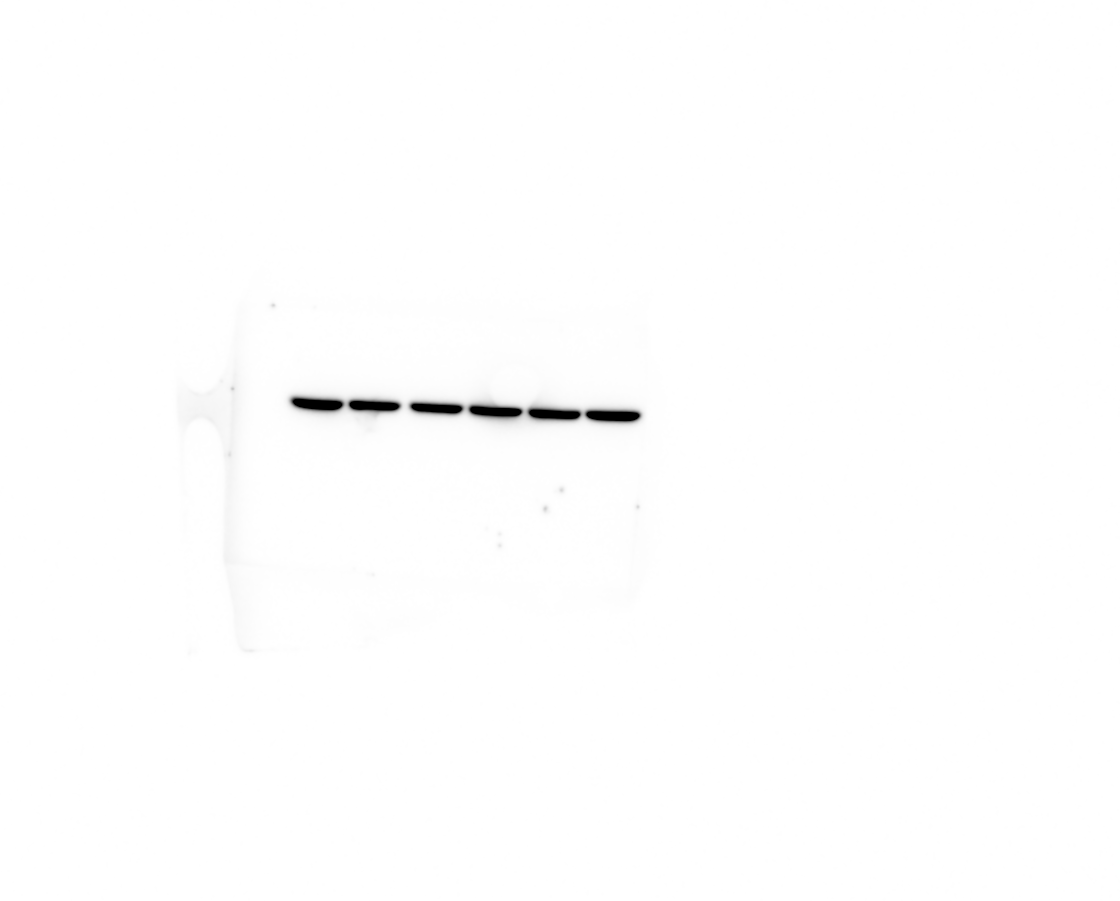

Supplement: Figure 5—source data 1. [file elife-78861-fig5-data1.zip › Figure 5D/Exp 2/Actin.tif]

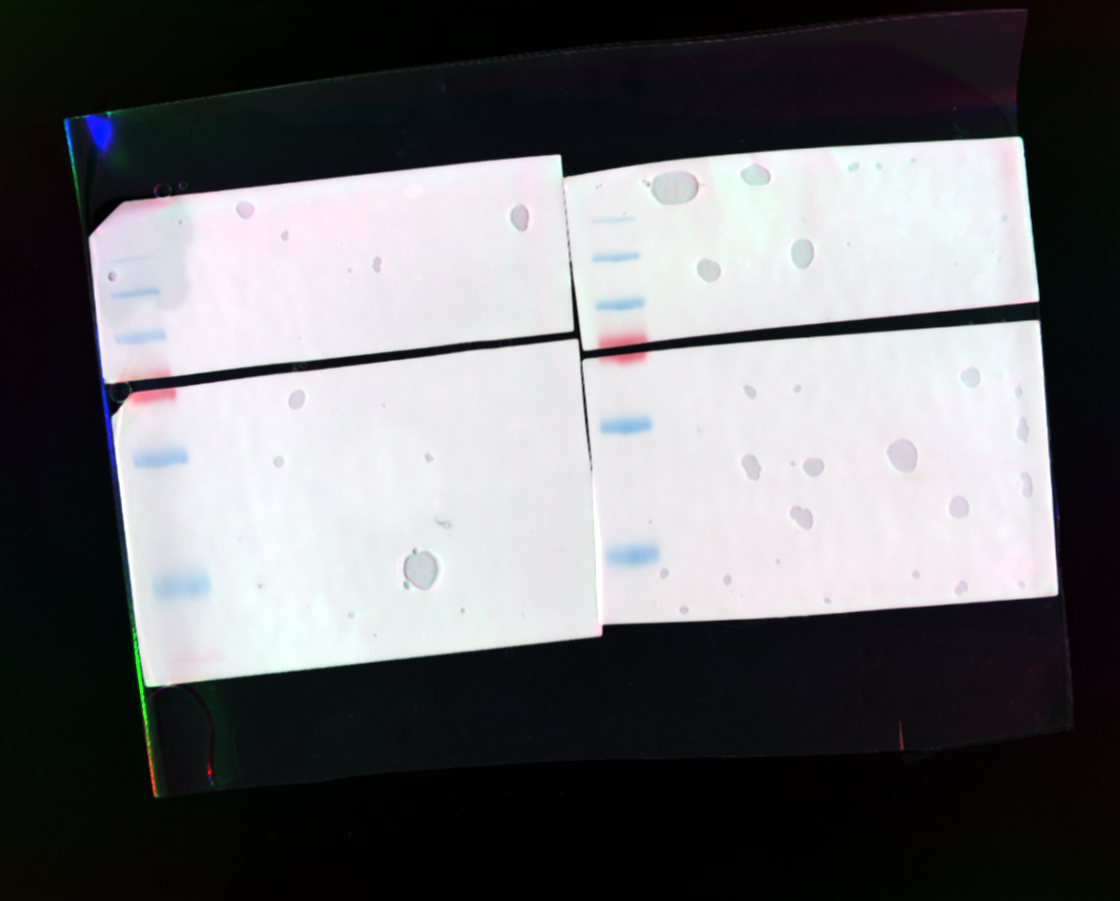

Supplement: Figure 5—source data 1. [file elife-78861-fig5-data1.zip › Figure 5D/Exp 2/Phospho-ERK Marker.tif]

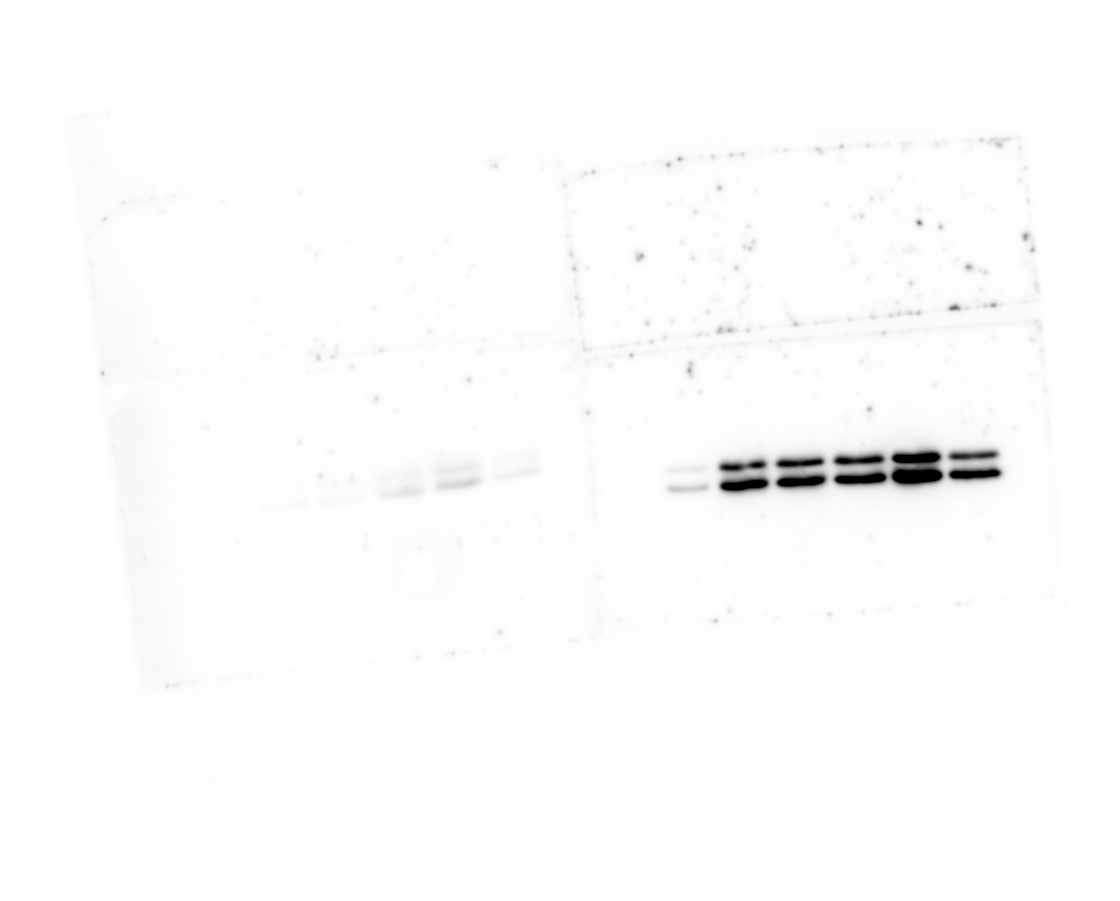

Supplement: Figure 5—source data 1. [file elife-78861-fig5-data1.zip › Figure 5D/Exp 2/Phospho-ERK.tif]

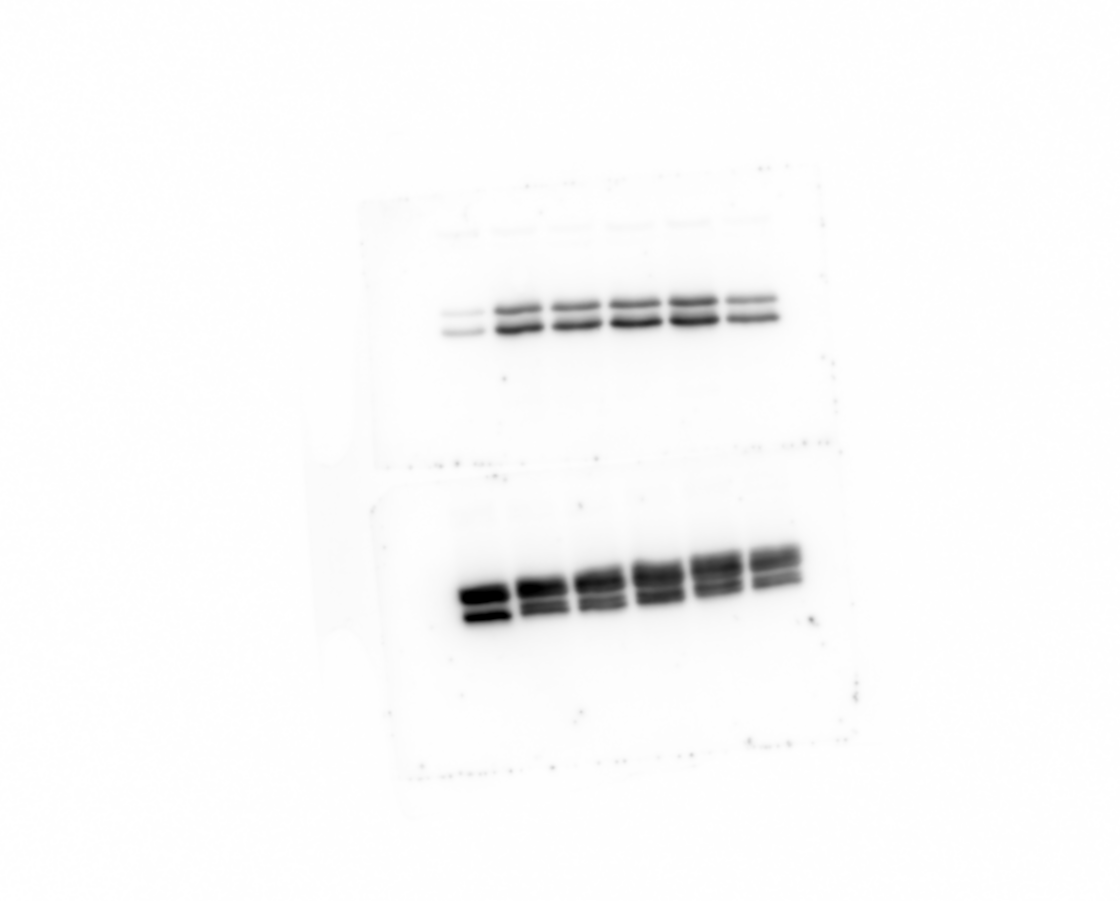

Supplement: Figure 5—source data 1. [file elife-78861-fig5-data1.zip › Figure 5D/Exp 2/Total ERK.tif]

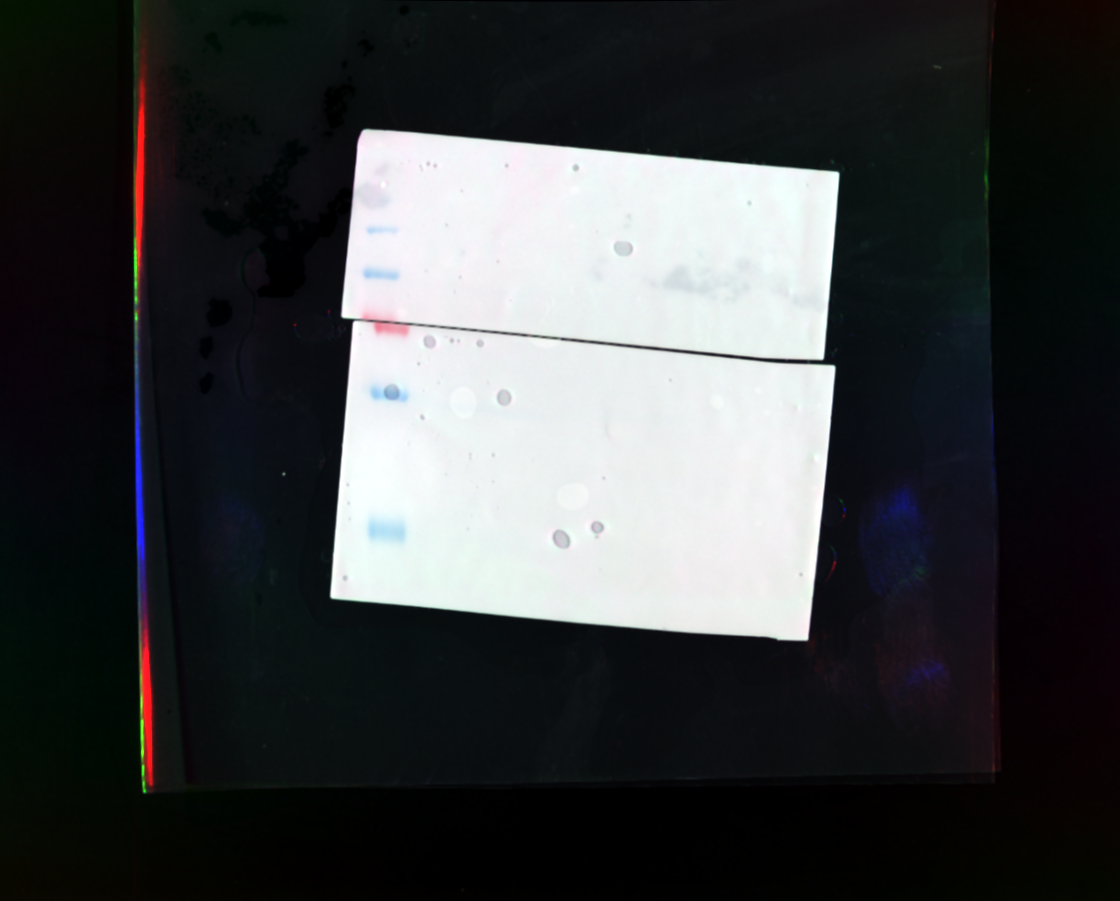

Supplement: Figure 5—source data 1. [file elife-78861-fig5-data1.zip › Figure 5D/Exp 1/Total ERK Marker.tif]

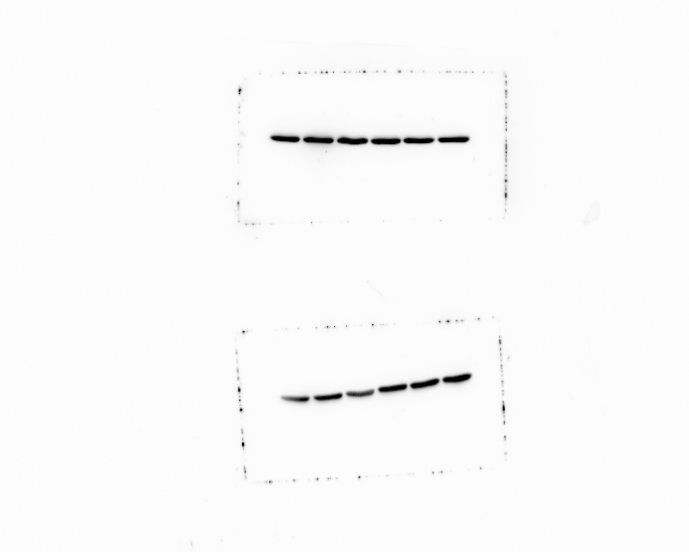

Supplement: Figure 5—source data 1. [file elife-78861-fig5-data1.zip › Figure 5D/Exp 1/Actin.tif]

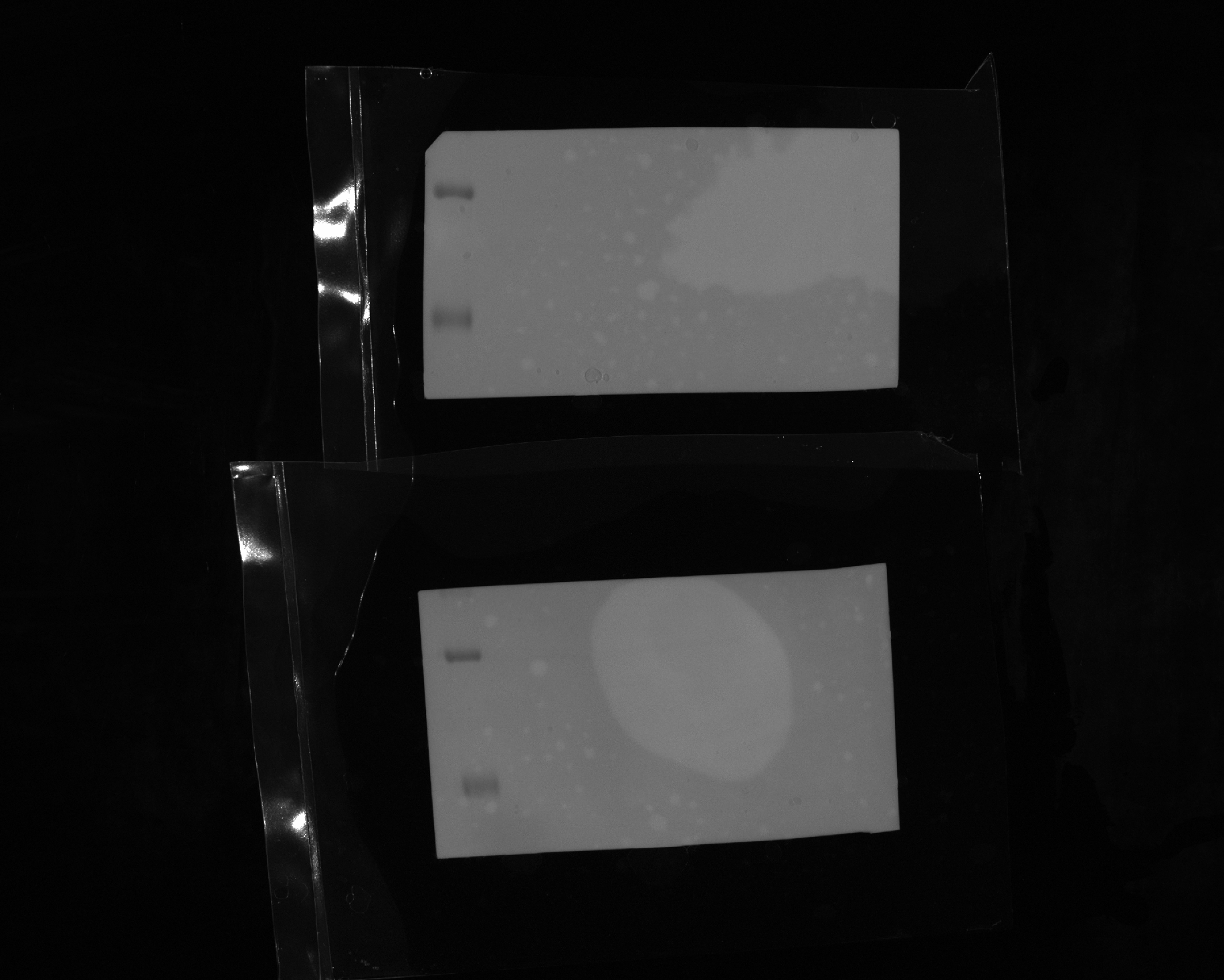

Supplement: Figure 5—source data 1. [file elife-78861-fig5-data1.zip › Figure 5D/Exp 1/Actin Marker.jpg]

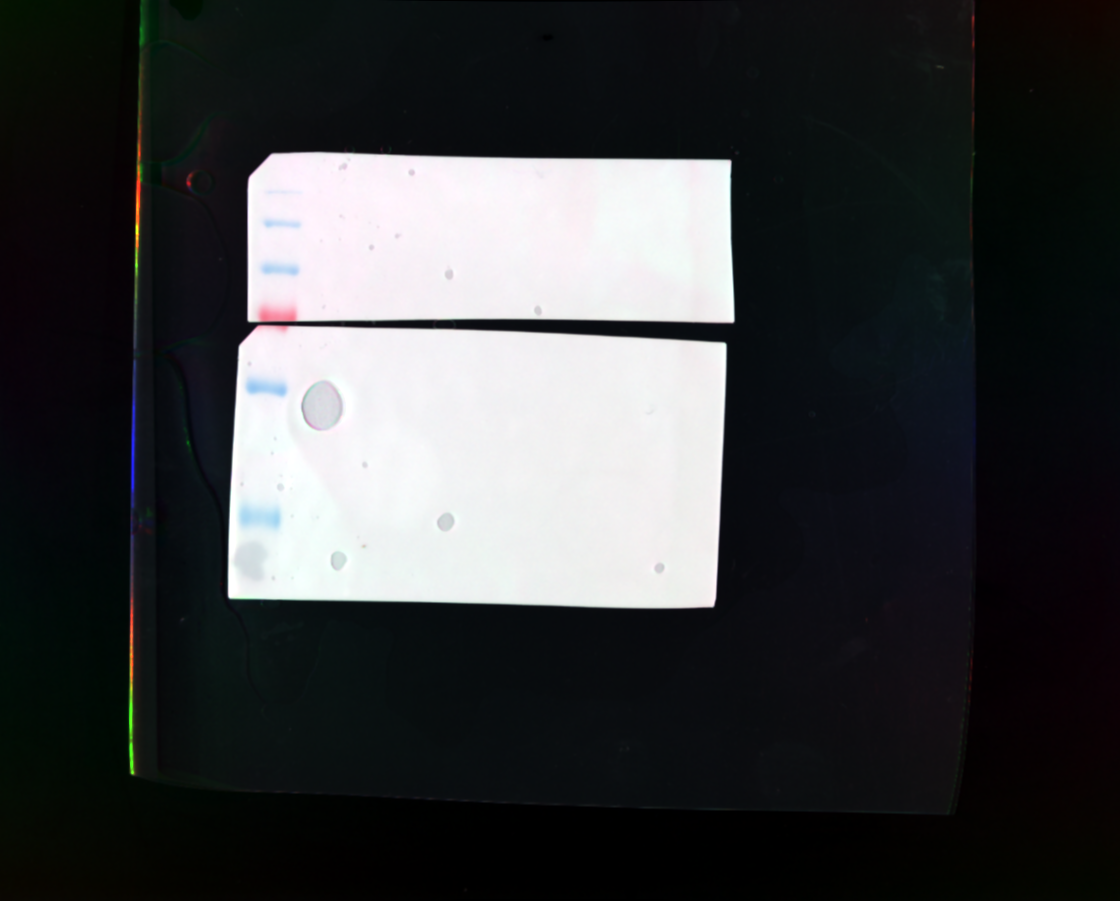

Supplement: Figure 5—source data 1. [file elife-78861-fig5-data1.zip › Figure 5D/Exp 1/Phospho-ERK Marker.tif]

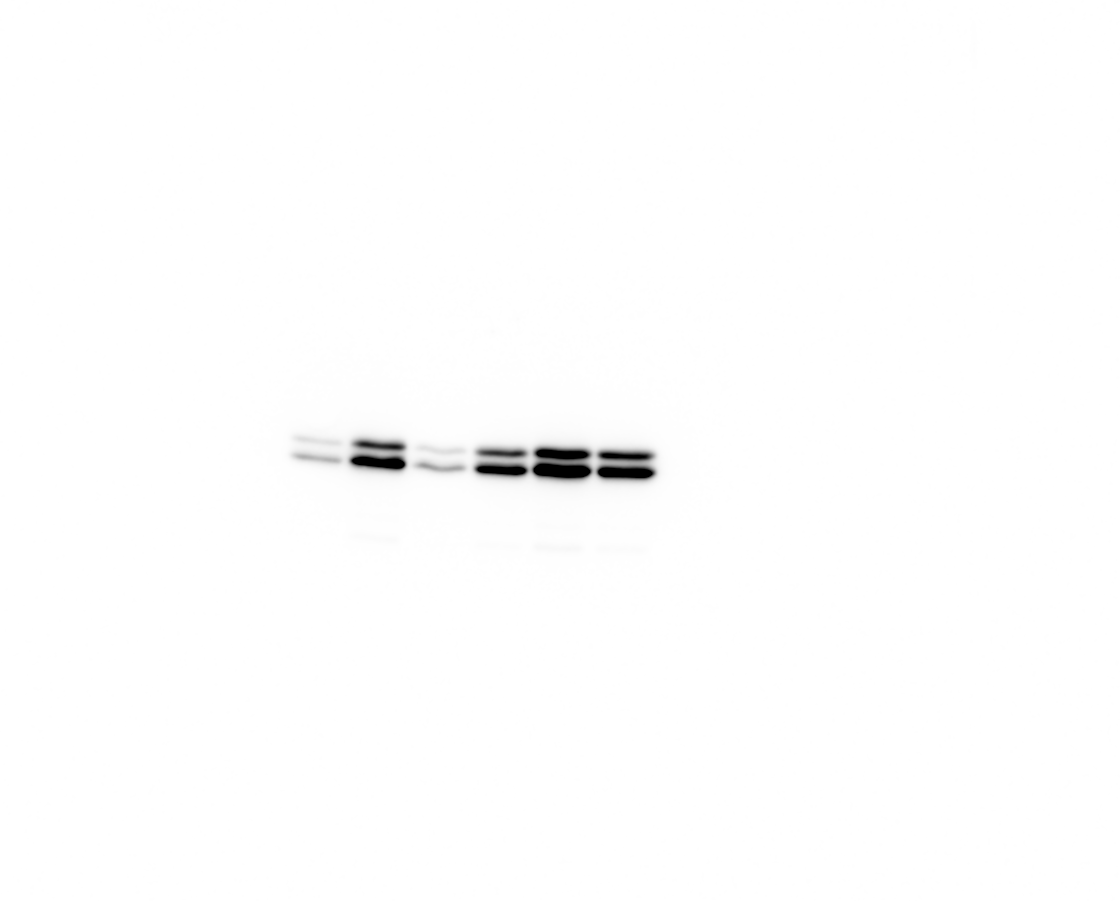

Supplement: Figure 5—source data 1. [file elife-78861-fig5-data1.zip › Figure 5D/Exp 1/Phospho-ERK.tif]

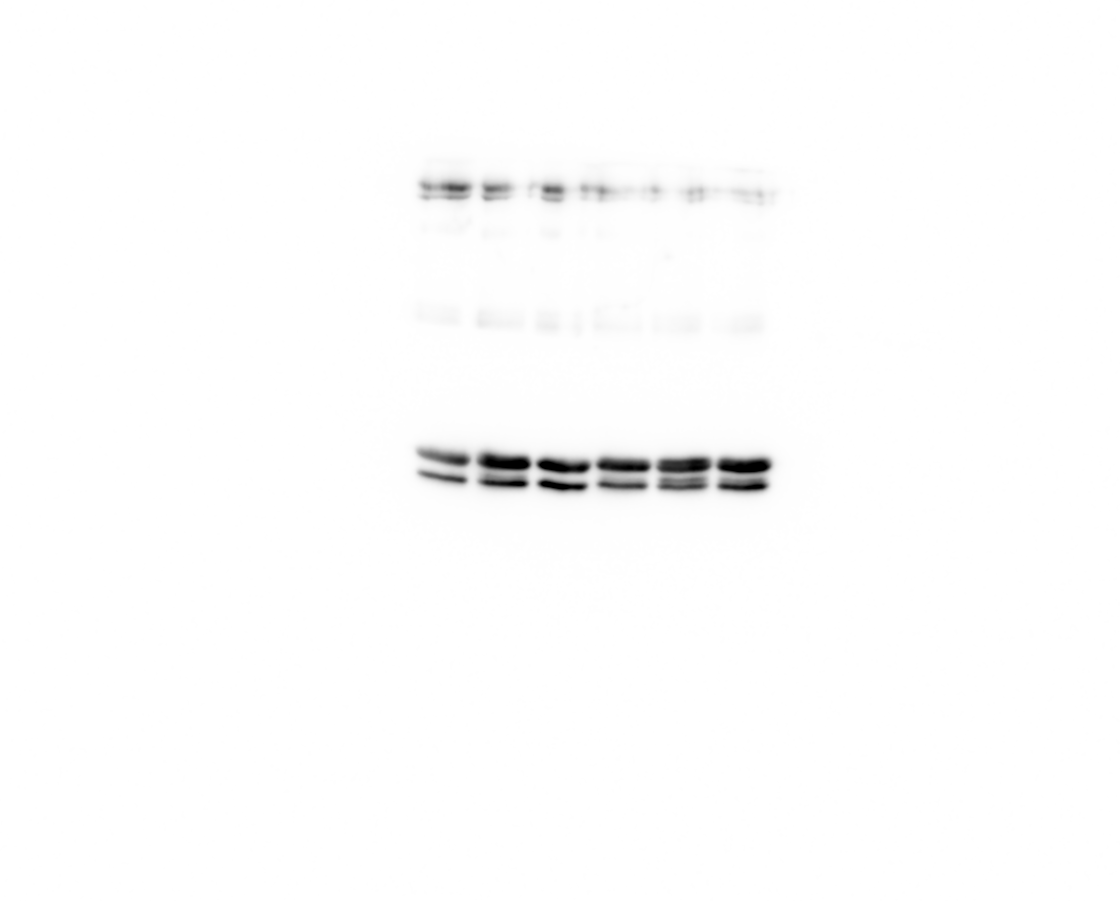

Supplement: Figure 5—source data 1. [file elife-78861-fig5-data1.zip › Figure 5D/Exp 1/Total ERK.tif]

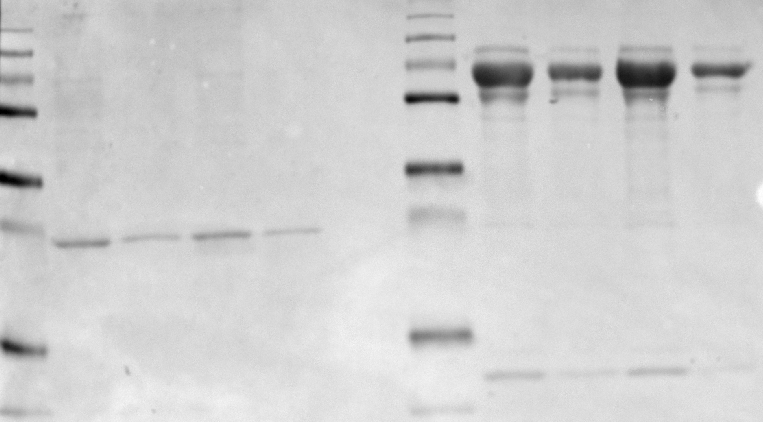

Supplement: Figure 7—figure supplement 1—source data 1. [file elife-78861-fig7-figsupp1-data1.zip › Figure 7 - Supplment 1 - Source Data/HDL and Protein - Coommassie staining Image.png]

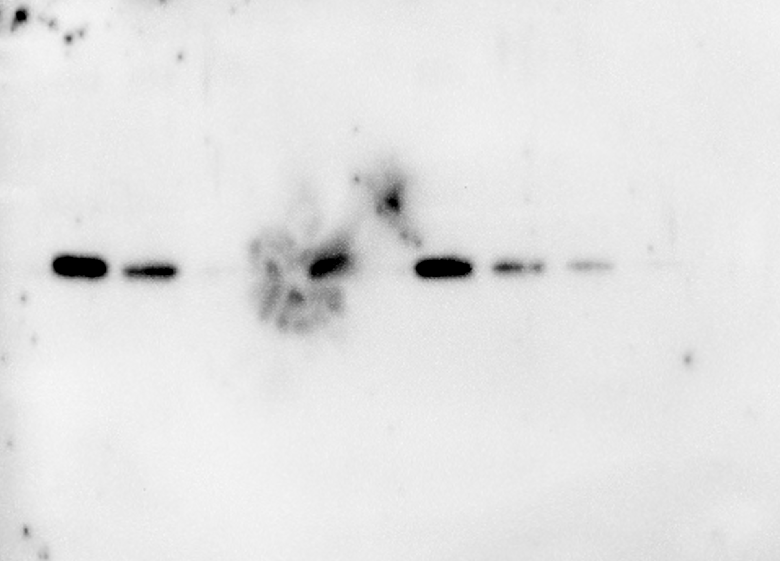

Supplement: Figure 7—figure supplement 1—source data 1. [file elife-78861-fig7-figsupp1-data1.zip › Figure 7 - Supplment 1 - Source Data/ApoM - Western Blotting Image.png]
