# Supplementary material for: Murine endothelial serine palmitoyltransferase 1 (SPTLC1) is required for vascular development and systemic sphingolipid homeostasis
Source: eLife. 2022 Oct 5;11:e78861. doi: 10.7554/eLife.78861 (PMC9578713; doi:10.7554/eLife.78861)
Supplement: Figure 1—figure supplement 2—source data 1. [file elife-78861-fig1-figsupp2-data1.zip › Figure 1 - Supplement 2 - Source Data/Blots with lane information.pptx]

## Slide 1
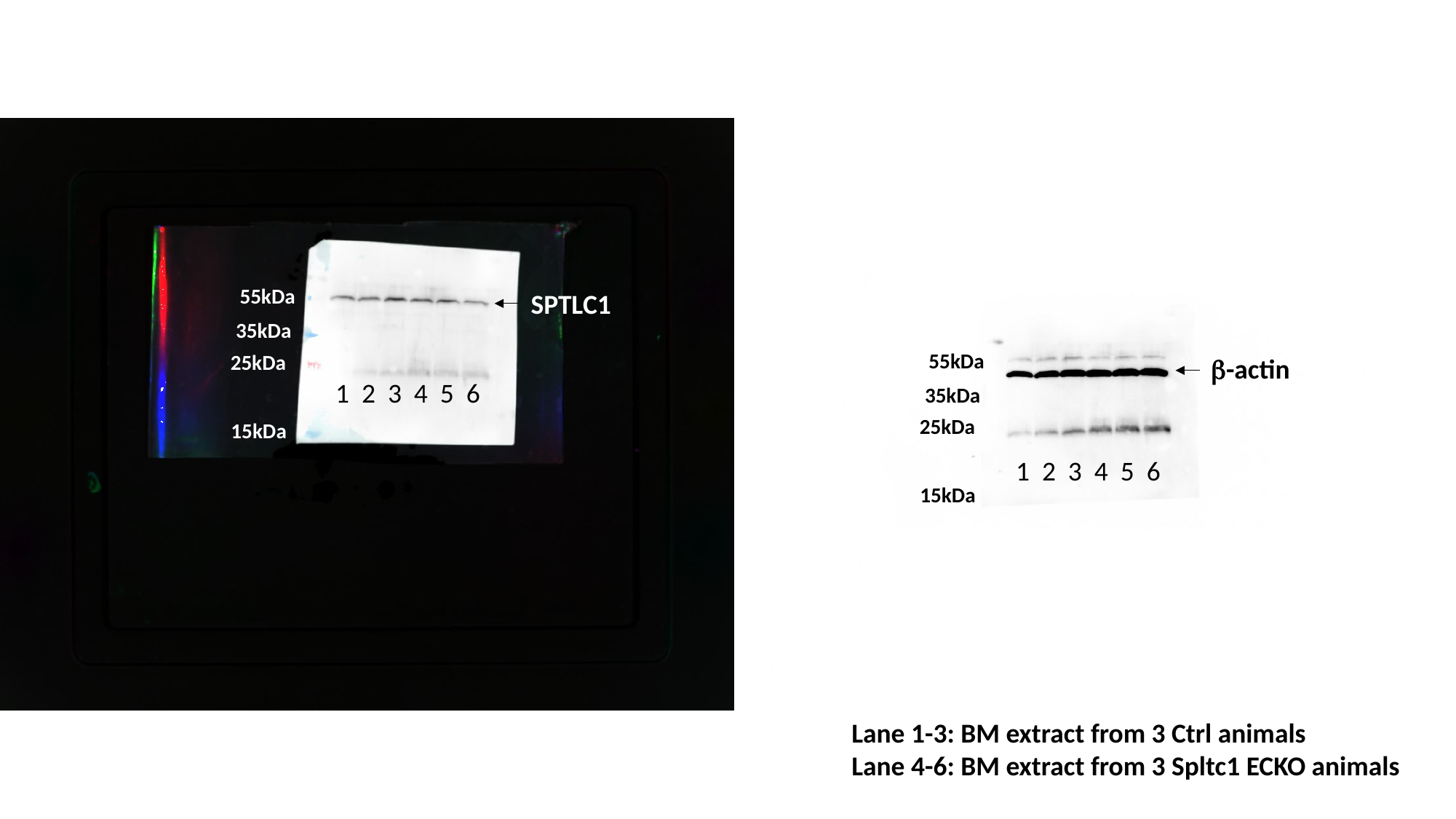

55kDa
SPTLC1
35kDa
55kDa
25kDa
b-actin
1 2 3 4 5 6
35kDa
25kDa
15kDa
1 2 3 4 5 6
15kDa
Lane 1-3: BM extract from 3 Ctrl animals
Lane 4-6: BM extract from 3 Spltc1 ECKO animals
