# Supplementary material for: Murine endothelial serine palmitoyltransferase 1 (SPTLC1) is required for vascular development and systemic sphingolipid homeostasis
Source: eLife. 2022 Oct 5;11:e78861. doi: 10.7554/eLife.78861 (PMC9578713; doi:10.7554/eLife.78861)
Supplement: Figure 1—figure supplement 4—source data 1. [file elife-78861-fig1-figsupp4-data1.zip › Figure 1 - Supplement 4/Blots with lane information.pptx]

## Slide 1
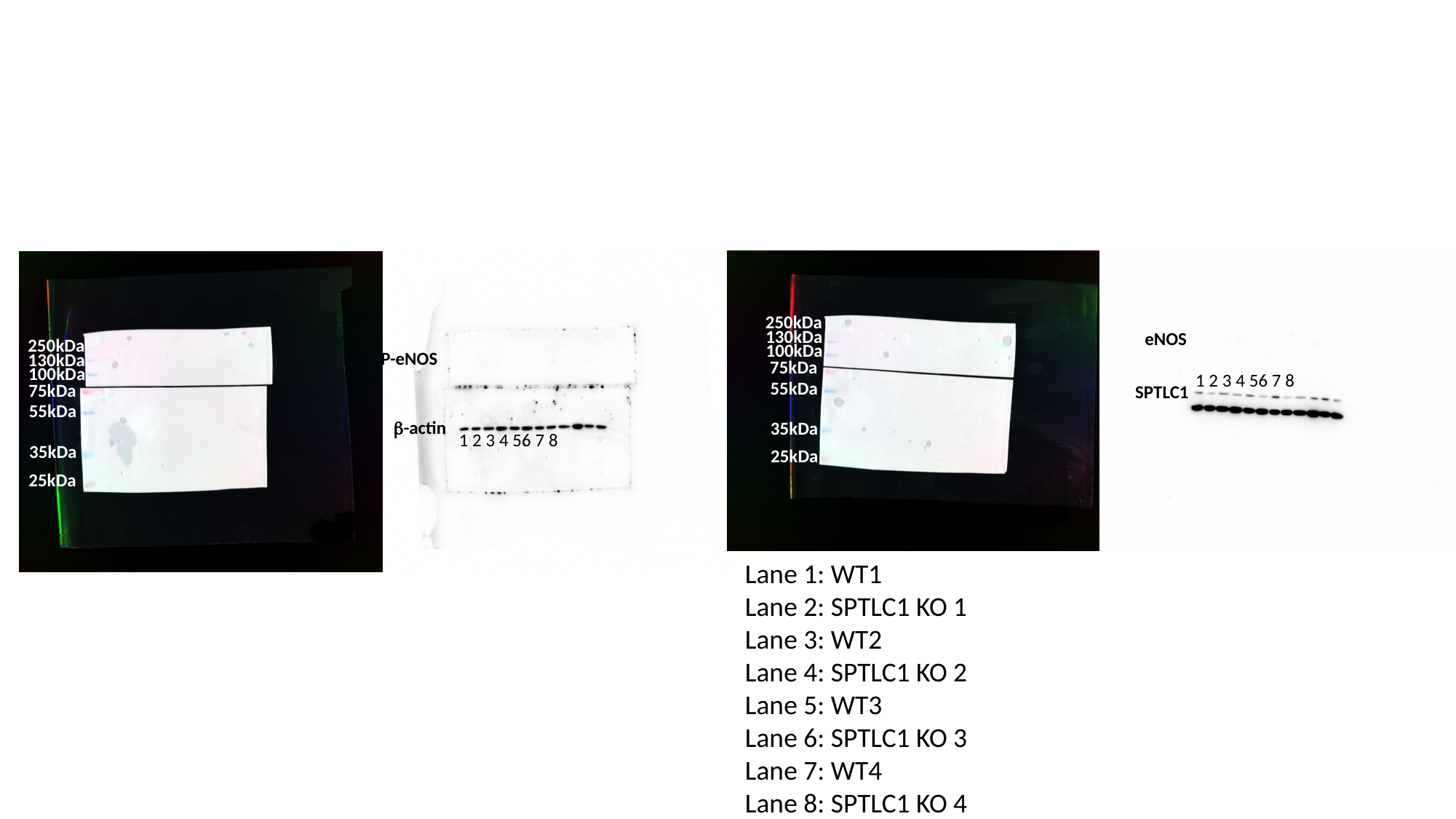

250kDa
130kDa
eNOS
250kDa
100kDa
P-eNOS
130kDa
75kDa
100kDa
1 2 3 4 56 7 8
55kDa
75kDa
SPTLC1
55kDa
b-actin
35kDa
1 2 3 4 56 7 8
35kDa
25kDa
25kDa
Lane 1: WT1
Lane 2: SPTLC1 KO 1
Lane 3: WT2
Lane 4: SPTLC1 KO 2
Lane 5: WT3
Lane 6: SPTLC1 KO 3
Lane 7: WT4
Lane 8: SPTLC1 KO 4
