# Supplementary material for: Murine endothelial serine palmitoyltransferase 1 (SPTLC1) is required for vascular development and systemic sphingolipid homeostasis
Source: eLife. 2022 Oct 5;11:e78861. doi: 10.7554/eLife.78861 (PMC9578713; doi:10.7554/eLife.78861)
Supplement: Figure 5—source data 1. [file elife-78861-fig5-data1.zip › Figure 5D/Blots with Lane Information.pptx]

## Slide 1
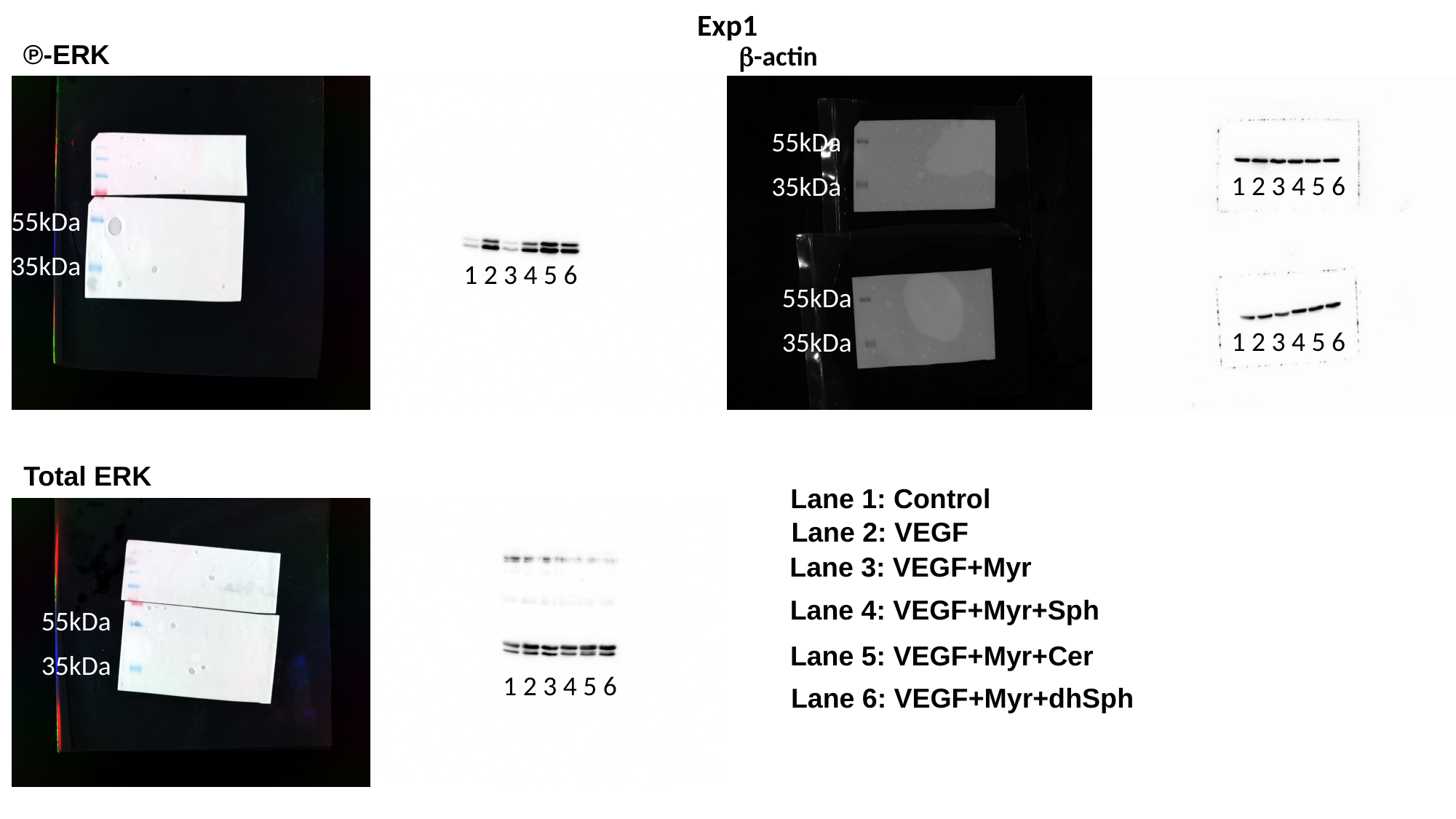

Exp1
℗-ERK
b-actin
55kDa
1 2 3 4 5 6
35kDa
55kDa
35kDa
1 2 3 4 5 6
55kDa
1 2 3 4 5 6
35kDa
Total ERK
Lane 1: Control
Lane 2: VEGF
Lane 3: VEGF+Myr
Lane 4: VEGF+Myr+Sph
55kDa
Lane 5: VEGF+Myr+Cer
35kDa
1 2 3 4 5 6
Lane 6: VEGF+Myr+dhSph

## Slide 2
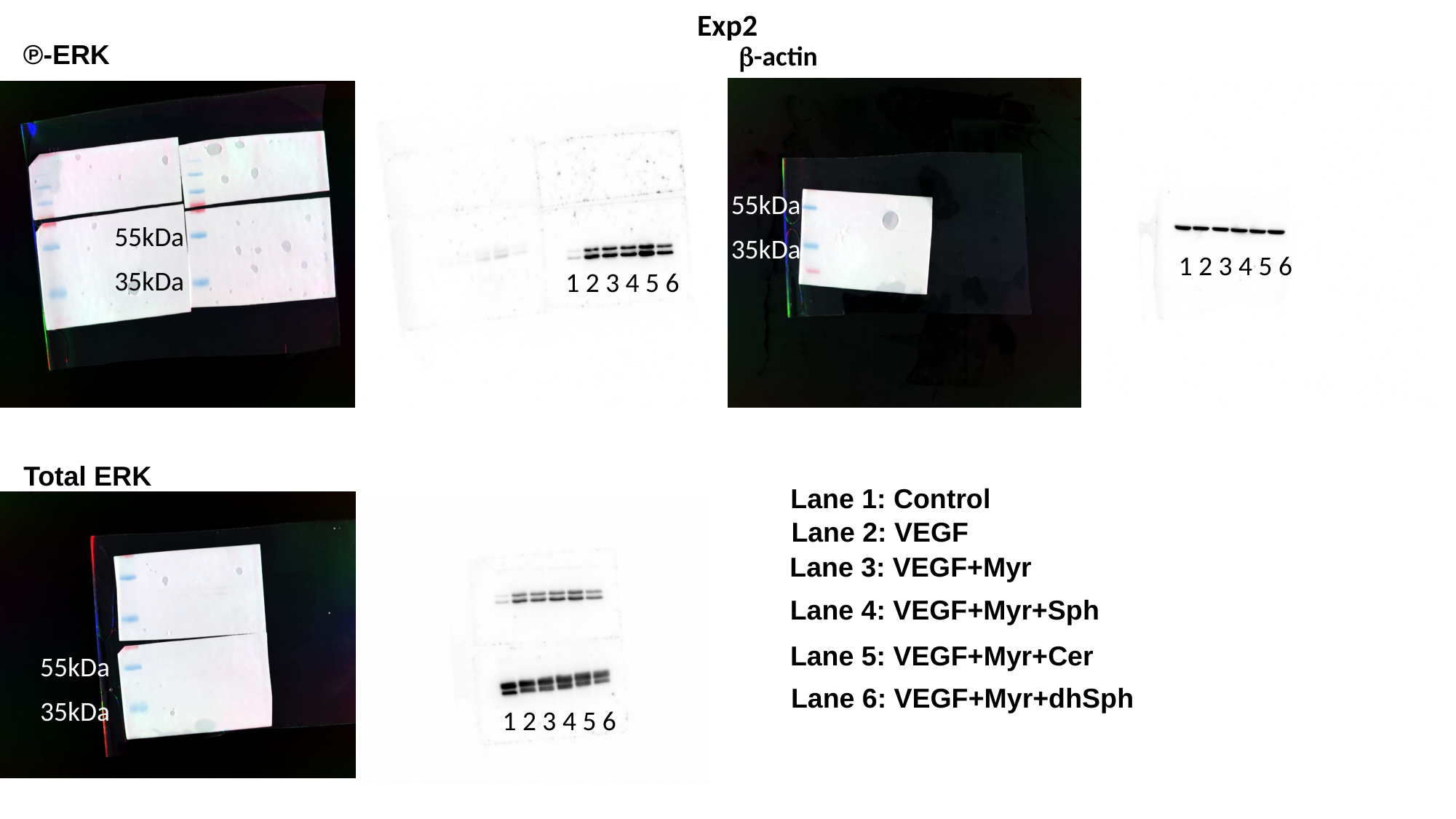

Exp2
℗-ERK
b-actin
55kDa
35kDa
55kDa
55kDa
55kDa
35kDa
1 2 3 4 5 6
35kDa
1 2 3 4 5 6
35kDa
1 2 3 4 5 6
55kDa
35kDa
Total ERK
Lane 1: Control
Lane 2: VEGF
Lane 3: VEGF+Myr
Lane 4: VEGF+Myr+Sph
55kDa
Lane 5: VEGF+Myr+Cer
35kDa
55kDa
Lane 6: VEGF+Myr+dhSph
35kDa
1 2 3 4 5 6

## Slide 3
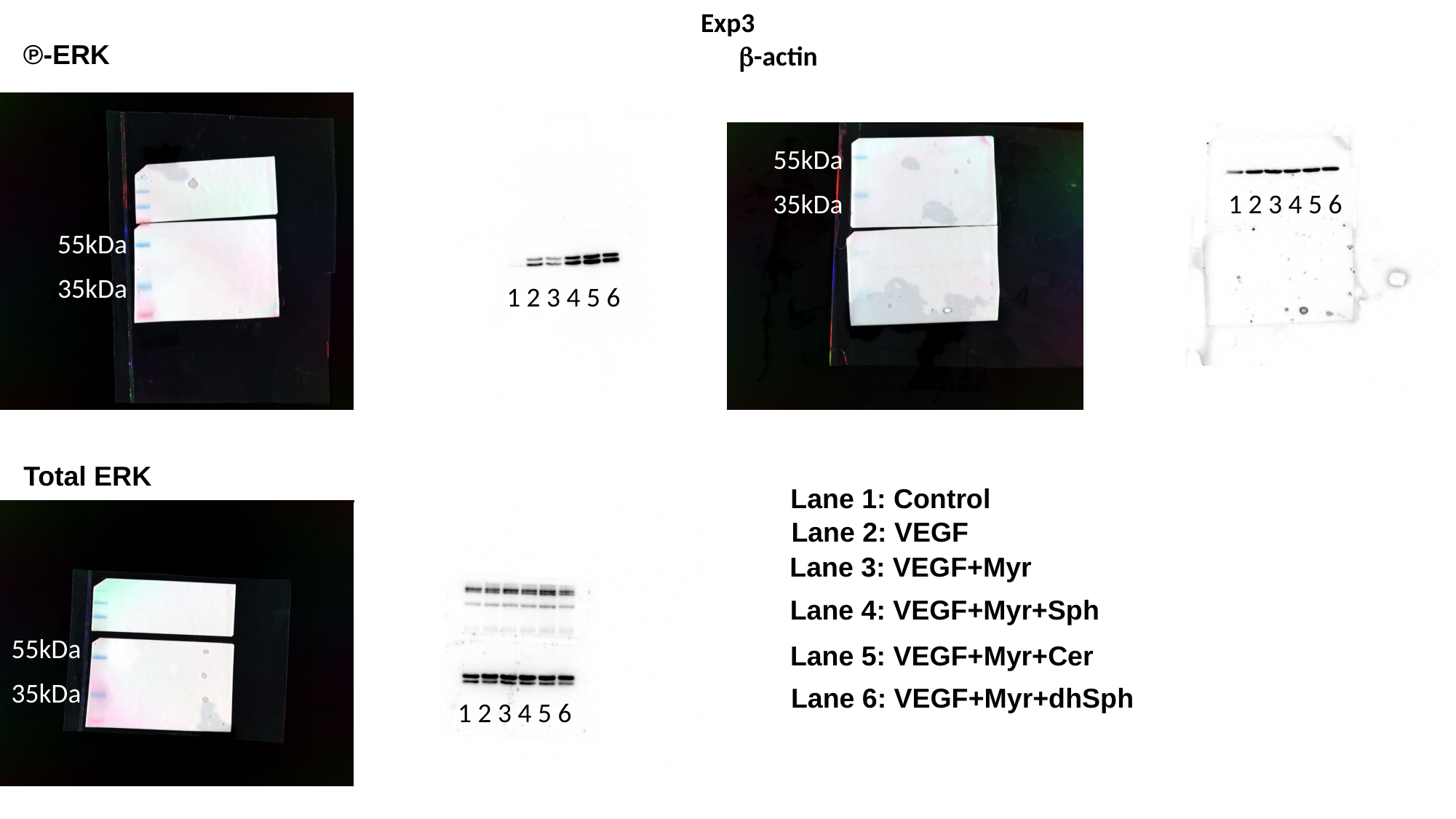

Exp3
℗-ERK
b-actin
55kDa
35kDa
1 2 3 4 5 6
55kDa
35kDa
1 2 3 4 5 6
Total ERK
Lane 1: Control
Lane 2: VEGF
Lane 3: VEGF+Myr
Lane 4: VEGF+Myr+Sph
55kDa
Lane 5: VEGF+Myr+Cer
35kDa
Lane 6: VEGF+Myr+dhSph
1 2 3 4 5 6

## Slide 4
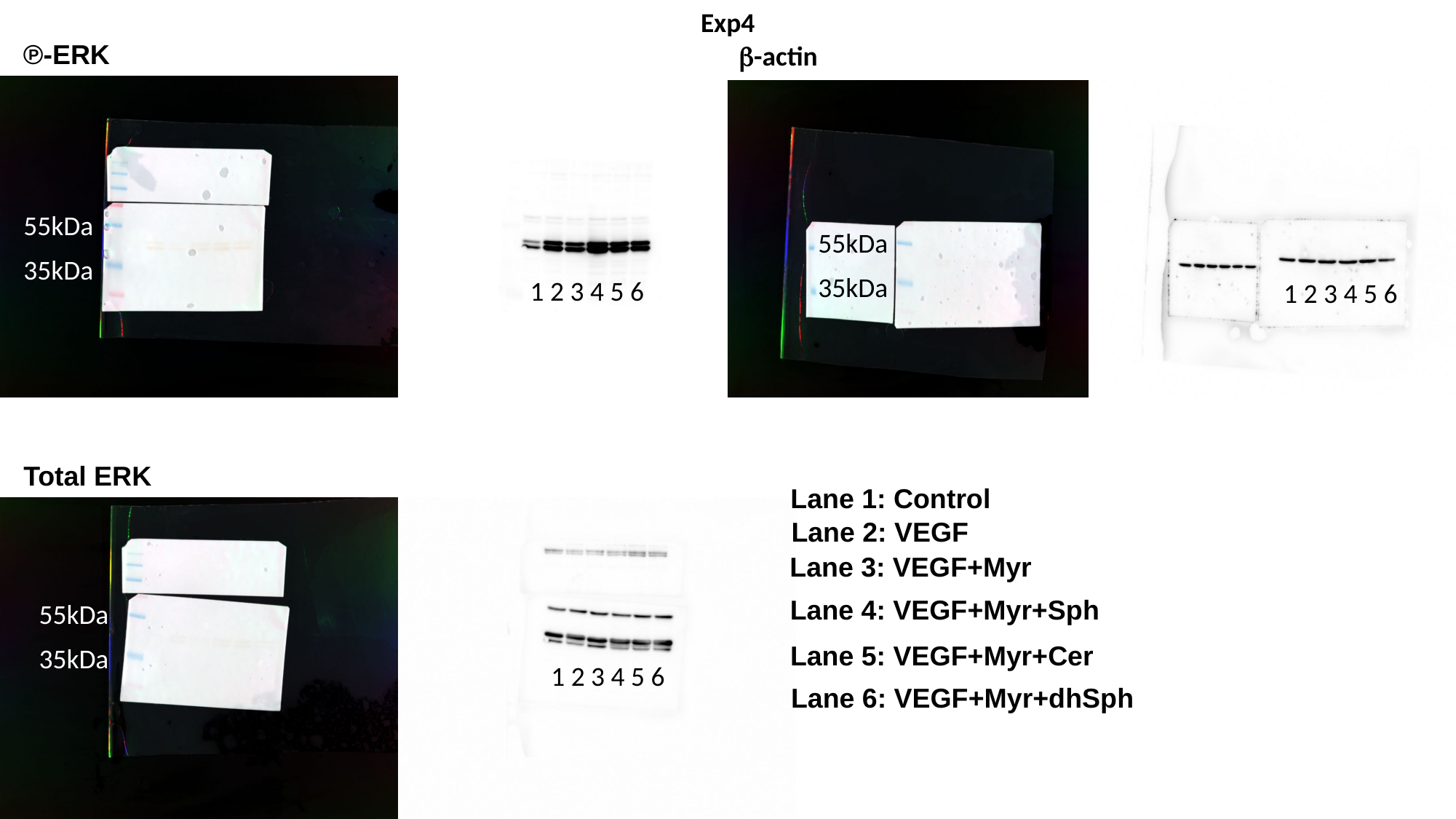

Exp4
℗-ERK
b-actin
55kDa
55kDa
35kDa
35kDa
1 2 3 4 5 6
1 2 3 4 5 6
Total ERK
Lane 1: Control
Lane 2: VEGF
Lane 3: VEGF+Myr
Lane 4: VEGF+Myr+Sph
55kDa
Lane 5: VEGF+Myr+Cer
35kDa
1 2 3 4 5 6
Lane 6: VEGF+Myr+dhSph
