# Supplementary material for: Murine endothelial serine palmitoyltransferase 1 (SPTLC1) is required for vascular development and systemic sphingolipid homeostasis
Source: eLife. 2022 Oct 5;11:e78861. doi: 10.7554/eLife.78861 (PMC9578713; doi:10.7554/eLife.78861)
Supplement: Figure 7—figure supplement 1—source data 1. [file elife-78861-fig7-figsupp1-data1.zip › Figure 7 - Supplment 1 - Source Data/Blots with lane information.pptx]

## Slide 1
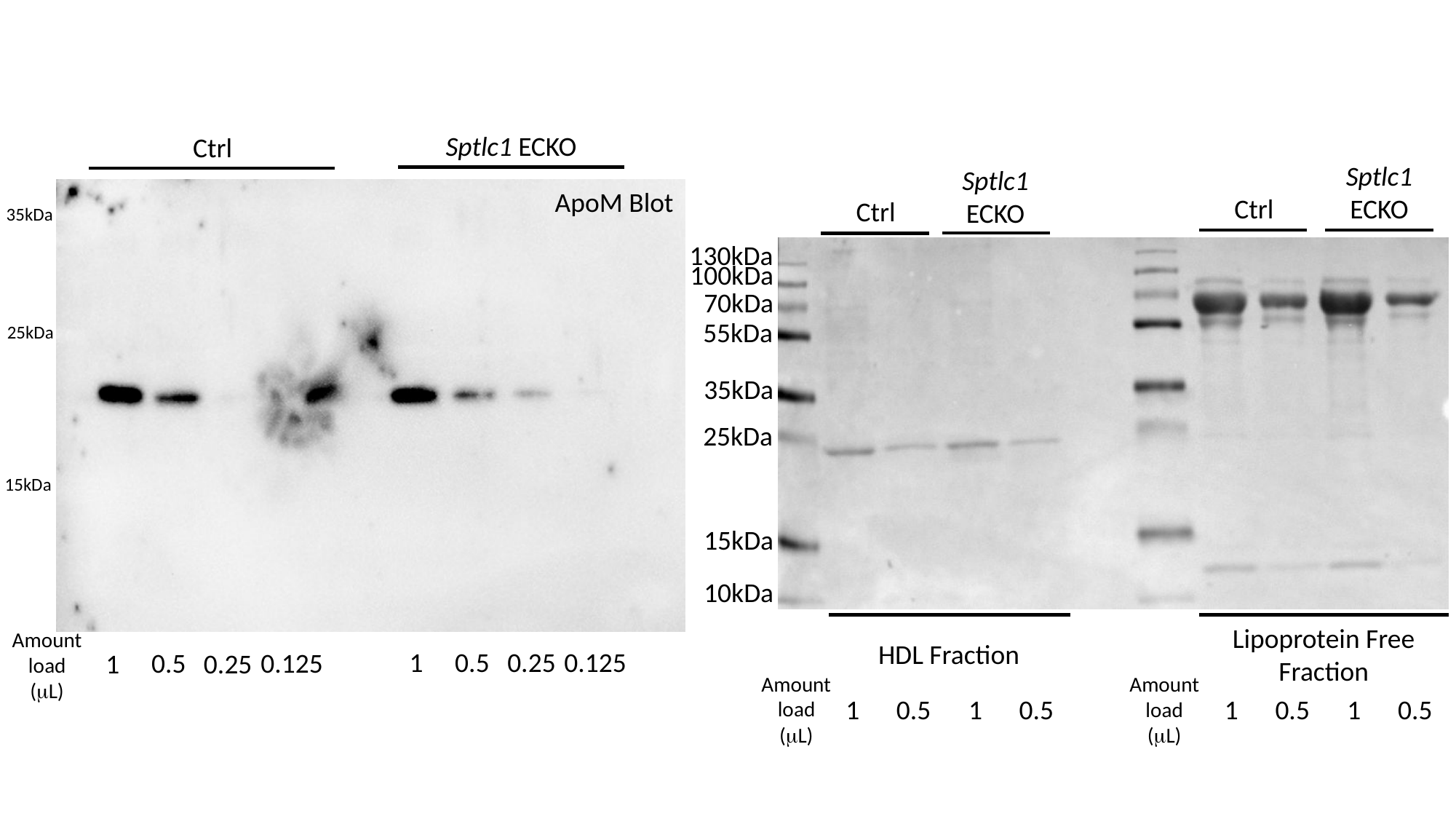

Sptlc1 ECKO
Ctrl
Sptlc1
ECKO
Sptlc1
ECKO
ApoM Blot
Ctrl
Ctrl
35kDa
130kDa
100kDa
70kDa
55kDa
25kDa
35kDa
25kDa
15kDa
15kDa
10kDa
Lipoprotein Free Fraction
Amount
load
(mL)
HDL Fraction
0.5
0.125
1
0.25
0.5
0.125
1
0.25
Amount
load
(mL)
Amount
load
(mL)
1
0.5
1
0.5
1
0.5
1
0.5
